# Robust Comparison of Raman Spectra Under Various Perturbations: Benchmarking Common Similarity Metrics and a New Peak-Oriented Index

**DOI:** 10.3390/mps9050148

**Published:** 2026-10-09

**Authors:** Adán Reyes-Reyes, Juan Andrés Reyes-Reyes, Erick Osvaldo Martínez-Ruíz, Alejandra Ortiz-Dosal, Ma. del Carmen Rodríguez-Aranda, Daniel Carlos Reyes-Reyes, Stiven Villada-Gil, Luis Carlos Ortiz-Dosal, Eleazar Samuel Kolosovas-Machuca

**Affiliations:** 1 Facultad de Ciencias, Universidad Autónoma de San Luis Potosí, Av. Parque Chapultepec 1570, San Luis Potosí 78295, Mexico; adan.reyes@uaslp.mx (A.R.-R.); a316156@alumnos.uaslp.mx (J.A.R.-R.); erick.martinez@uaslp.mx (E.O.M.-R.); alejandra.ortiz@uaslp.mx (A.O.-D.); carmen.aranda@uaslp.mx (M.d.C.R.-A.); 2 Coordinación para la Innovación y la Aplicación de la Ciencia y la Tecnología (CIACYT), Universidad Autónoma de San Luis Potosí, Av. Sierra Leona 550, San Luis Potosí 78210, Mexico; 3 Secretaría de Ciencia, Humanidades, Tecnología e Innovación, Universidad Autónoma de San Luis Potosí, Av. Parque Chapultepec 1570, San Luis Potosí 78295, Mexico; 4 Facultad de Ingeniería, Universidad Autónoma de San Luis Potosí, Av. Dr. Manuel Nava 304, Zona Universitaria, San Luis Potosí 78210, Mexico; daniel.reyes@uaslp.mx; 5 Facultad de Ciencias y Educación, Politécnico Colombiano Jaime Isaza Cadavid, Medellín 050022, Colombia; svillada@elpoli.edu.co; 6 Maestría en Ciencia e Ingeniería de los Materiales (MCIM-UAZ), Universidad Autónoma de Zacatecas, 801 López Velarde St. 9800, Zacatecas 98160, Mexico; ortiz.dosal.lc@uaz.edu.mx

**Keywords:** Raman spectroscopy, spectral similarity, robustness, perturbations, data augmentation, peak matching

## Abstract

Matching Raman spectra is complicated by shifts in band position, changes in intensity and linewidth, the appearance or loss of bands, and noise because different similarity measures preserve different forms of spectral evidence. We introduce $S_{J}$, a peak-oriented index based on one-to-one band assignment and explicit penalties for unmatched peaks, and evaluate it together with pointwise, rigid-alignment, elastic-alignment, and derivative-based measures. The benchmark comprises 500 baseline-corrected reference spectra and 50,000 controlled perturbed queries. Each noisy query was independently converted to a peak table before $S_{J}$ evaluation. Parameter sensitivity was assessed on 350 calibration references, after which the selected $S_{J}$ configuration and a single hybrid-ranking weight were fixed for 150 held-out references. The moderate association between $S_{J}$ and the waveform metrics ($\rho_{s}=0.28$–0.48) indicates that band correspondence retains information not captured by waveform agreement alone. In full-library retrieval, standalone $S_{J}$ attained a held-out Top-1 recovery of 0.9603. Combining $S_{J}$ with cosine similarity, Pearson correlation, cross-correlation, or cross-cosine similarity using the same calibration-selected weight (w=0.50) increased their respective Top-1 recovery rates from 0.4166 to 0.8828, 0.3960 to 0.9094, 0.9449 to 0.9930, and 0.9523 to 0.9933; reference-level bootstrap intervals for all four changes excluded zero. These results establish $S_{J}$ as a complementary structural layer that can strengthen both shift-sensitive and shift-tolerant library rankings, rather than as a universal replacement for waveform metrics.

## 1. Introduction

Raman spectroscopy [1] provides molecular fingerprints widely used across materials science and applied spectroscopy [2,3,4,5]. In many operational workflows, a measured spectrum is compared to reference spectra (or libraries) to produce similarity scores used for ranking, identification, and quality control. Such practice routinely relies on correlation/hit quality index (HQI)-style matching or closely related measures, including applied identification, mixture search, and instrument-aware library-search scenarios [6,7,8,9,10,11].

In practice, however, spectra acquired across instruments, sessions, or experimental conditions often deviate from the “directly comparable vector” assumption. Background fluorescence and baseline drift, calibration imperfections and interpolation, optical alignment, and acquisition settings can induce baseline distortions, Raman-shift offsets, intensity scaling, and changes in apparent linewidth or peak shape [12,13,14,15,16]. These effects can strongly bias pointwise metrics such as Pearson correlation and cosine similarity (spectral angle): even small shifts can cause peak-to-shoulder comparisons and rapidly reduce pointwise agreement [12,17,18,19]. Consequently, robustness depends on artifact type and strength, as well as on spectral morphology and preprocessing choices, which vary across instruments and application domains.

To mitigate misalignment, alignment-aware strategies are often used, including lag-optimized cross-correlation and nonlinear alignment approaches such as dynamic time warping (DTW) and its Raman-specific variants [20,21]. Nevertheless, shift handling alone does not address structural differences such as peak disappearance, appearance of spurious peaks, or local peak-shape changes. This motivates robustness-focused evaluation: instead of reporting a single similarity value on a static dataset, one should quantify how similarity measures respond to controlled perturbations that emulate realistic variability. In addition, although many similarity measures have been proposed across signal processing and chemometrics, it is nontrivial to validate them comprehensively across the diversity of Raman morphologies and measurement artifacts encountered in practice. For this reason, studies often focus on a subset of widely used measures that represent distinct design philosophies (pointwise, shift-aware, and peak/feature-oriented), while providing a benchmark structure that can be extended as additional measures are incorporated [12,17,18].

The present study adopts an augmentation-driven robustness benchmark grounded in a peak-table representation. Each reference spectrum is first mapped to a multi-peak representation, then used to synthesize multiple perturbed realizations that combine smooth correlated fields for shift, gain, and full width at half maximum (FWHM), discrete peak-level events (dropping, mutation, injection), and controlled Gaussian noise. The benchmark asks a practical question: after a reference spectrum is exposed to mixed perturbations, what kind of spectral evidence does each metric still capture? We therefore compare each original spectrum against its perturbed variants using pointwise, alignment-aware, elastic-alignment, and peak-oriented measures, and we interpret the results as a multi-layer decision problem. The controlled design allows “stress-testing” of similarity scores under artifact families that frequently co-occur in real sensing workflows and that are difficult to isolate in purely observational datasets.

In addition to pointwise and alignment-based metrics, we report a peak-oriented similarity index that compares band parameters rather than raw pointwise traces. Peak-based and Raman-specific approaches are increasingly used in practical library-search contexts, for example by fitting peaks (including Voigt/Lorentzian models), emphasizing peak-bearing regions, comparing relative intensity relations between characteristic peaks, or introducing domain-specific similarity functions for mixture-oriented search [10,22,23,24]. Our implementation relies on Lorentzian peak models and minimum-total-cost one-to-one peak pairing via the Hungarian algorithm [25,26,27].

We place the proposed index next to several existing Raman-comparison families. Segmental HQI and voting strategies modify how spectral evidence is aggregated for library search [7,9]; Raman-specific kernel or weighted-distance metrics encode local spectral neighborhoods and mixture-oriented intensity conditions [10]; squared first-difference Euclidean cosine (SFEC) and related mathematical scores test different waveform-level invariances [18]; Characteristic Peak Similarity (CaPSim) and approaches based on Relative Intensity Information between Characteristic Peaks (RIICP) use characteristic peak regions or relative intensity information between characteristic peaks to improve Raman/surface-enhanced Raman scattering (SERS) identification workflows [24,28]; and peak-fitting studies show that fitted band parameters depend on line-shape assumptions, model selection, and weak-band recovery [22,23,29]. The present benchmark brings these ideas into a pairwise robustness setting by asking how direct, shift-tolerant, elastic, derivative, and peak-correspondence evidence change when the same Raman reference spectrum is exposed to controlled mixed perturbations. Experiments are conducted on a heterogeneous public spectral collection rather than on a single database, combining mineral, planetary/materials, open-library, and synthetic Raman sources [30,31,32]. The Methods section therefore keeps waveform-level comparison separate from peak-oriented band correspondence and introduces $S_{J}$ as an inspectable structural layer. Table 1 summarizes how these representative method families relate to the diagnostic role of the present benchmark.

A direct numerical comparison across these external peak-aware families would require validated, method-specific preprocessing, peak extraction, characteristic-region definitions, and calibration against a common evaluation target. We therefore restrict the numerical claims to the measures implemented in this study; the other families provide methodological context and define priorities for future cross-method validation.

The study makes four contributions. It provides a reproducible benchmark of controlled Raman perturbations across 500 references and 50,000 queries. It compares metrics that represent direct waveform agreement, rigid and elastic alignment, and explicit band correspondence within the same perturbation design. It separates parameter selection from evaluation by choosing four $S_{J}$ scoring tolerances and one global hybrid weight on grouped calibration references before held-out testing. Finally, it provides an online implementation [36] in which the Lorentzian decomposition can be inspected, adjusted, or replaced by an external peak table before $S_{J}$ is evaluated. The contribution is evaluated through perturbation response and complementary retrieval performance, without treating $S_{J}$ as a replacement for every existing similarity method.

The remainder of the paper is organized as follows. Section 2 describes the collection, assumptions, benchmark design, peak-table construction, perturbations, similarity measures, parameter calibration, and retrieval analysis. Section 3 reports fit quality, parameter sensitivity, perturbation response, metric complementarity, and held-out library retrieval. Section 4 summarizes the accompanying application programming interface (API). Supplementary Material S1 provides the extended algorithms, complete calibration and uncertainty results, retrieval details, an illustrated API quick-start, and instructions for inspecting or replacing the automatic peak table.

## 2. Materials and Methods

### 2.1. Datasets from Public Raman Databases

The benchmark comprises Raman reference spectra from explicitly identified public repositories and a documented synthetic generator. The public/reference subset originates from the RRUFF Raman mineral database [30], the National Aeronautics and Space Administration (NASA) Raman Spectroscopic Database (RAMdb) [31], the OpenSpecy library data [37], and the Raman Open Database [38]; the synthetic subset was generated with RamanSPy [32]. Together, these records broaden the controlled stress test across spectral morphologies and source types; they are not treated as an estimate of source-specific experimental variability. For the benchmark reported in this paper, the final reference set contains S=500 spectra. Each reference spectrum is paired with R=100 perturbed realizations, yielding 50,000 reference–perturbed comparisons per similarity metric.

The 500-spectrum set combines 249 public experimental or reference spectra and 251 RamanSPy-generated synthetic spectra. The synthetic records extend the range of documented spectral morphologies and band arrangements but are not treated as experimental acquisitions. Table 2 reports the composition of the collection and the identity-level calibration and held-out partition. All 100 perturbations derived from one reference remain in the same partition.

Available source metadata were retained to delimit and document the collection. Depending on the original provider, these fields include the database and record identifiers, source uniform resource locator (URL), spectral range, excitation wavelength, material description, temperature, instrument model, power, resolution, polarization, and chemical formula. Physical state, sample form, and grain size were not reported consistently across the contributing repositories; these attributes were therefore neither inferred nor used as statistical covariates. Other missing acquisition conditions were likewise left unspecified. The traceability record preserves the available fields, explicitly marks missing values, distinguishes synthetic RamanSPy entries from experimental records, and reports each spectrum’s sampled Raman-shift range. The complete collection is available through Zenodo [39].

### 2.2. Similarity Measures and Evidence Domains

Let $y_{a},y_{b}\in R^{n}$ be two spectra on the same grid. The measures below represent complementary evidence domains: direct pointwise agreement, rigid-shift recovery, elastic alignment, derivative agreement, and peak-level correspondence. Because each measure encodes different invariances to scaling, offsets, or alignment, their robustness differs under the controlled perturbations used in this study [12,33].

#### 2.2.1. Pearson Correlation

The Pearson correlation index between $y_{a}$ and $y_{b}$ is

(1) $$\rho \left(y_{a},y_{b}\right)=\frac{\sum_{i=1}^{n}\left(y_{a,i}-{\bar{y}}_{a}\right)\left(y_{b,i}-{\bar{y}}_{b}\right)}{\sqrt{\sum_{i=1}^{n}{\left(y_{a,i}-{\bar{y}}_{a}\right)}^{2}}\sqrt{\sum_{i=1}^{n}{\left(y_{b,i}-{\bar{y}}_{b}\right)}^{2}}},\;{\bar{y}}_{a}=\frac{1}{n}\sum_{i=1}^{n}y_{a,i}.$$

Pearson correlation measures linear covariation after mean removal [18]. It is invariant to global affine intensity transformations, which is advantageous when absolute scaling is unstable. However, it remains strictly pointwise: intensities are compared at fixed $x_{i}$. Small Raman-shift offsets can therefore compare a peak maximum in one spectrum with a shoulder or baseline point in the other and rapidly reduce agreement [12,33].

#### 2.2.2. Cosine Similarity and Spectral Angle

Cosine similarity and the corresponding spectral angle are

(2) $$\cos\left(y_{a},y_{b}\right)=\frac{\langle y_{a},y_{b}\rangle }{{∥y_{a}∥}_{2}{∥y_{b}∥}_{2}},\;\theta =\arccos\left(\cos\left(\cdot \right)\right).$$

Cosine similarity compares the angle between spectral vectors and is invariant to global multiplicative gain. Unlike Pearson correlation, it does not center the data, so residual baselines can influence the dot product. It also remains sensitive to misalignment because shifted peaks reduce pointwise overlap [12,19].

#### 2.2.3. Normalized Cross-Correlation with Limited Shift

The normalized cross-correlation index is

(3) $$r_{\mathrm{xcorr}}=\max_{\ell \in [-L,L]}\frac{\sum_{i}\left(y_{a,i}-{\bar{y}}_{a}\right)\left(y_{b,i+\ell }-{\bar{y}}_{b,\ell }\right)}{\sqrt{\sum_{i}{\left(y_{a,i}-{\bar{y}}_{a}\right)}^{2}}\sqrt{\sum_{i}{\left(y_{b,i+\ell }-{\bar{y}}_{b,\ell }\right)}^{2}}}.$$

The admissible rigid-shift bound was fixed at $L=25\;{\mathrm{cm}}^{-1}$ and implemented as the corresponding integer lag window on the common resampled grid. Cross-correlation can recover agreement after a bounded global offset, but it cannot correct nonrigid distortions or directly account for missing or additional bands.

#### 2.2.4. Lag-Optimized Cosine Similarity (Cross-Cosine)

Cross-cosine preserves the angular criterion while allowing the same bounded rigid displacement:

(4) $$s_{\mathrm{xcos}}=\max_{\ell \in [-L,L]}\frac{\sum_{i}y_{a,i}y_{b,i+\ell }}{\sqrt{\sum_{i}y_{a,i}^{2}}\sqrt{\sum_{i}y_{b,i+\ell }^{2}}}.$$

This waveform-level measure indicates whether similarity improves after an allowed rigid displacement. It does not provide explicit evidence about matched, missing, or additional bands.

#### 2.2.5. Dynamic Time Warping (DTW)

The DTW-based similarity used here is defined by

(5) $$\mathrm{DTW}\left(i,j\right)={\left(y_{a,i}-y_{b,j}\right)}^{2}+\min\left\{\mathrm{DTW}\left(i-1,j\right),\mathrm{DTW}\left(i,j-1\right),\mathrm{DTW}\left(i-1,j-1\right)\right\},$$

(6) $$d_{\mathrm{dtw}}=\sqrt{\frac{\mathrm{DTW}(n,n)}{L_{\mathrm{path}}}},\;S_{\mathrm{dtw}}=\exp\;\left(-\frac{d_{\mathrm{dtw}}}{\sigma_{\mathrm{ref}}+\epsilon_{0}}\right),\;\epsilon_{0}>0.$$

DTW permits nonlinear alignment through a monotone warping path. This flexibility can accommodate local displacement patterns, but it may also produce unintuitive agreement when noise or baseline variation dominates or when bands disappear or appear.

#### 2.2.6. Squared First-Difference Euclidean Cosine (SFEC)

For first differences,

(7) $$\Delta y_{a,i}=y_{a,i+1}-y_{a,i},\;\Delta y_{b,i}=y_{b,i+1}-y_{b,i},\;i=1,\ldots ,n-1,$$

and the SFEC score is

(8) $$\mathrm{SFEC}\left(y_{a},y_{b}\right)={\left(\frac{\langle \Delta y_{a},\Delta y_{b}\rangle }{{∥\Delta y_{a}∥}_{2}{∥\Delta y_{b}∥}_{2}}\right)}^{2}.$$

SFEC compares local spectral slopes and suppresses constant offsets. Differentiation, however, amplifies high-frequency noise and becomes less robust when broadening flattens the slopes [18].

#### 2.2.7. Peak-Oriented Similarity (Proposed Index, $S_{J}$)

The peak-oriented similarity index $S_{J}\in \left[0,1\right]$ compares band parameters rather than sampled intensities. Although this benchmark uses Lorentzian peak tables for reproducibility, the score requires only consistently defined peak centers, normalized heights, and widths. It proceeds in three conceptual stages: represent each spectrum by a peak table (Supplementary Section S1.1), obtain a minimum-total-cost one-to-one correspondence through Hungarian assignment with explicit orphans (Supplementary Section S1.2), and combine match quality and structural differences into a normalized overlap score inspired by soft Jaccard and F-measure principles [40].

Let the reference peak set be $P^{*}={\left\{\left(x_{i}^{*},y_{i}^{*},F_{i}^{*}\right)\right\}}_{i=1}^{n}$ and the query peak set be $P={\left\{\left(x_{j},y_{j},F_{j}\right)\right\}}_{j=1}^{m}$, where *x* is the peak center (cm^−1^), y∈[0,1] is the normalized peak height, and *F* is the FWHM (cm^−1^). Each spectrum is internally scaled so that its maximum peak height is one before $S_{J}$ is evaluated. Deviations are mapped to bounded penalties using

(9) $$s_{k}\left(\Delta \right)=\tanh^{2}\;\left(\frac{k\Delta }{2}\right),$$

with *k* chosen so that $s_{k}\left(\tau \right)=0.5$ for its corresponding tolerance τ. Thus, τ is a soft half-penalty scale, not an acceptance threshold. Every nonzero discrepancy receives a penalty, and deviations above τ remain admissible while their penalties increase smoothly toward saturation.

Two tolerance sets have separate roles. Matching tolerances $\left(\tau_{x}^{H},\tau_{y}^{H}\right)$ define the Hungarian assignment costs, whereas metric tolerances $\left(\tau_{x},\tau_{y},\tau_{F}\right)$ grade the selected matches. Using weights $(\alpha_{x},\alpha_{y})\geq 0$, the assignment cost between reference peak *i* and query peak *j* is

(10) $$C_{ij}=\alpha_{x}s_{k_{x}^{H}}\left(\right|x_{i}^{*}-x_{j}\left|\right)+\alpha_{y}s_{k_{y}^{H}}\left(\right|y_{i}^{*}-y_{j}\left|\right).$$

A physical gate sets $C_{ij}\leftarrow M$ whenever $|x_{i}^{*}-x_{j}|\;>\Delta x_{\max}$, with M≫1. Dummy assignments with costs $U_{\mathrm{ref}}$ and $U_{\mathrm{qry}}$ permit unmatched bands. Solving the augmented assignment problem yields the matched set MA and the orphan sets $U_{\mathrm{ref}}$ and $U_{\mathrm{qry}}$.

For each matched pair (i,j)∈MA, the average discrepancy and match quality are

(11) $$e_{ij}=\frac{1}{M_{ij}}\left[s_{k_{x}}\left(\right|x_{i}^{*}-x_{j}\left|\right)+s_{k_{y}}\left(\right|y_{i}^{*}-y_{j}\left|\right)+s_{k_{F}}\left(\right|F_{i}^{*}-F_{j}\left|\right)\right],\;m_{ij}=1-e_{ij}\in \left[0,1\right],$$

where $M_{ij}$ is the number of included attributes. With positive reference-side weights $\omega_{i}$ normalized over the matched reference set, the effective true-positive mass is

(12) $${\mathrm{TP}}_{\mathrm{eff}}=\sum_{(i,j)\in \mathrm{MA}}\omega_{i}m_{ij}.$$

Uniform weights are used throughout the present benchmark. The definition and implementation permit alternative weights, but diagnostic-band and region-specific weighting strategies are not evaluated here.

Orphan peaks contribute effective false-negative and false-positive terms,

(13) $${\mathrm{FN}}_{\mathrm{eff}}=\frac{1}{\max(|\mathrm{MA}|,1)}\sum_{i\in U_{\mathrm{ref}}}s_{k_{y}^{\mathrm{non}}}\left(\Delta y_{i}\right),\;{\mathrm{FP}}_{\mathrm{eff}}=\frac{1}{\max(|\mathrm{MA}|,1)}\sum_{j\in U_{\mathrm{qry}}}s_{k_{y}^{\mathrm{non}}}\left(\Delta y_{j}\right),$$

where $k_{y}^{\mathrm{non}}$ corresponds to an orphan-severity scale $\tau_{y}^{\mathrm{non}}$. The final quality-aware soft Jaccard score is

(14) $$\begin{array}{c} \;S_{J}=\frac{{\mathrm{TP}}_{\mathrm{eff}}}{1+{\mathrm{FN}}_{\mathrm{eff}}+{\mathrm{FP}}_{\mathrm{eff}}}\in \left[0,1\right]\; \end{array},$$

with $S_{J}=0$ when |MA|=0. The score therefore combines the quality of paired bands with explicit penalties for missing and additional bands.

The peak-table construction and the score are distinct operations. The automatic Lorentzian procedure described below supplies one reproducible preliminary table, but $S_{J}$ can instead use bands obtained by expert curation, validated peak-picking software, instrument software, or another suitable line-shape model. The Hungarian matches, orphan sets, and per-band discrepancies are also available as inspectable outputs rather than being hidden inside the scalar score. Algorithm 1 summarizes the complete peak-oriented similarity calculation.
**Algorithm 1** Peak-oriented similarity pipeline: peak table, Hungarian assignment, and soft Jaccard score**Require:** Reference and query peak tables with tuples (x,y,F). **Require:** Assignment weights $\left(\alpha_{x},\alpha_{y}\right)$, matching tolerances $\left(\tau_{x}^{H},\tau_{y}^{H}\right)$, scoring tolerances $\left(\tau_{x},\tau_{y},\tau_{F}\right)$, orphan-severity scale $\tau_{y}^{\mathrm{non}}$, center gate $\Delta x_{\max}$, and orphan costs $U_{\mathrm{ref}},U_{\mathrm{qry}}$. **Ensure:** Matches MA, orphan sets $U_{\mathrm{ref}},U_{\mathrm{qry}}$, per-pair qualities, and $S_{J}\in \left[0,1\right]$.
1:Normalize peak heights within each table.2:Construct the pairwise cost matrix using Equation (10) and apply the center gate.3:Augment the matrix with dummy assignments and solve the Hungarian assignment problem.4:Compute the matched-pair qualities using Equation (11) and ${\mathrm{TP}}_{\mathrm{eff}}$ using Equation (12).5:Compute ${\mathrm{FN}}_{\mathrm{eff}}$ and ${\mathrm{FP}}_{\mathrm{eff}}$ using Equation (13).6:Return $S_{J}$ using Equation (14).

### 2.3. Study Questions, Assumptions, and Analysis Plan

The study addresses two sequential questions. First, how do the comparison principles summarized above respond to the same controlled changes in Raman spectra? Second, can the band-correspondence information supplied by $S_{J}$ complement full-library rankings produced by waveform measures? The metrics are therefore interpreted according to the evidence they retain, rather than ordered as candidates for one universally best score.

Two input assumptions delimit the benchmark. The spectra are assumed to have undergone baseline correction and to be ready for spectral comparison and peak-table construction; fluorescence and baseline variability are consequently outside the numerical claims. In addition, $S_{J}$ requires a peak table containing band centers, relative heights, and width descriptors. The Lorentzian procedure used here is one automated way to obtain such a table, not part of the definition of $S_{J}$. Tables produced by expert annotation, validated peak picking, instrument software, or another suitable line-shape model can be used instead. Supplementary Sections S1.1 and S1.2 describe the automated route and assignment procedure.

Reference identities are partitioned into calibration and held-out groups, with all 100 perturbations of a reference kept together. Four $S_{J}$ scoring tolerances are examined and selected on calibration references; the remaining assignment settings and uniform peak weighting are fixed and reported. The selected configuration is then frozen for the perturbation analysis. In the library task, every query is compared with all 500 references by each waveform selector and by $S_{J}$. One global hybrid weight is selected on calibration references and applied to the complete 500-reference ranking of every held-out query. Reference-level block bootstrap preserves dependence among perturbations of the same reference. Supplementary Sections S1.3–S1.6 provide the complete calibration, perturbation, retrieval, and uncertainty outputs.

### 2.4. Pipeline Overview

**Figure 1:**
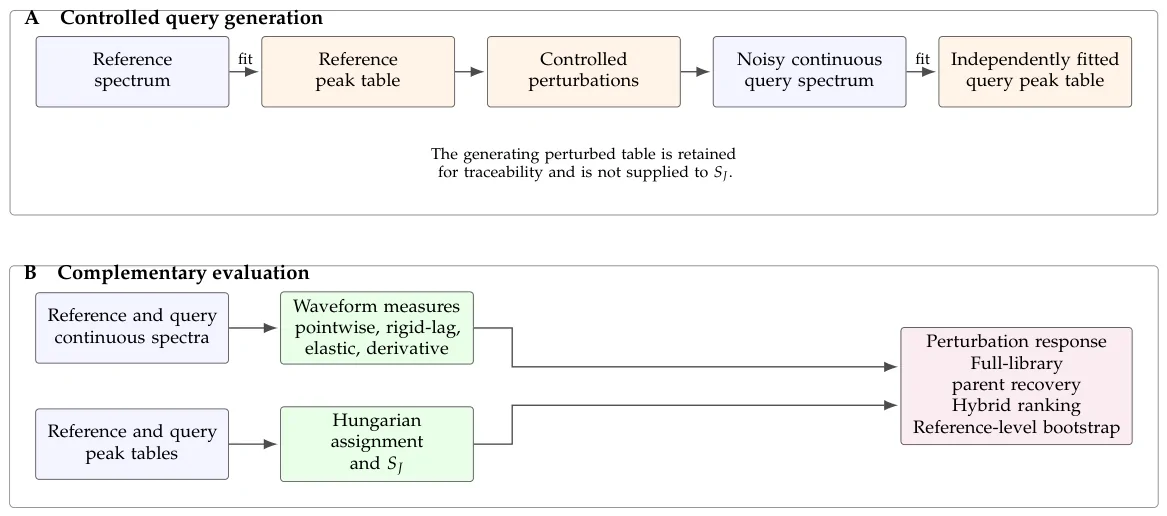
Robustness-benchmark workflow. (**A**) Controlled perturbations are used to synthesize a noisy query, which is independently decomposed before $S_{J}$ is evaluated. The generating perturbed table is retained for traceability but is not supplied to the score. (**B**) Waveform measures and $S_{J}$ evaluate the same query through continuous-spectrum and peak-table representations, respectively, before perturbation-response, full-library retrieval, hybrid-ranking, and reference-level uncertainty analyses.

Figure 1 summarizes the benchmark structure. A reference spectrum is represented by a peak table, from which a controlled perturbed table and continuous spectrum are generated. Gaussian noise is added to the continuous-query spectrum, and a new peak table is then estimated from that noisy query. Waveform metrics compare the reference and noisy continuous spectra, whereas $S_{J}$ compares the reference table with the independently estimated noisy-query table. Thus, $S_{J}$ does not receive the hidden perturbed table used to generate the query.

Notation convention used in this section: index *s* denotes the sample identity, and index *r* denotes the perturbation realization. We use r=0 for the reference reconstruction and r=1,…,R for perturbed realizations.

For each reference spectrum $y_{s}\left(x\right)$, we first construct a peak-consistent reference spectrum $y_{s,0}\left(x\right)$ by fitting a multi-Lorentzian model and reconstructing the signal from the resulting peak table $P_{s,0}$ (Supplementary Section S1.1). In this design, the source spectrum acts as the empirical anchor, while $y_{s,0}\left(x\right)$ and $P_{s,0}$ are the controlled reference objects used in the calculations. The Lorentzian fit provides a reproducible way to build this benchmark. The $S_{J}$ score itself begins after peak-table construction and can be applied to peak tables obtained from expert curation, validated peak-picking routines, instrument software, or other line-shape models appropriate to the target application.

Perturbed spectra ${\left\{y_{s,r}\left(x\right)\right\}}_{r=1}^{R}$ are then generated by modifying peak parameters through correlated smooth fields (shift, gain, and broadening) and discrete peak-level events (dropping, mutation, and injection), followed by the addition of Gaussian noise at a prescribed signal-to-noise ratio (SNR). The full perturbation model is described in Section 2.6.

For each realization, similarity is evaluated between the reference and perturbed spectra,

(15) $$S_{s,r}=S\left(y_{s,0},\;y_{s,r}\right),$$

where S denotes one of the candidate similarity metrics. The grouped calibration partition is used to select the four $S_{J}$ scoring tolerances and one hybrid-ranking weight; these settings are then fixed for the held-out analysis. Repeating the procedure across all spectra and perturbations yields the metric-response, full-library retrieval, and reference-level bootstrap summaries reported in Section 3.

We also define a controlled reference-library retrieval test using the same benchmark objects. Each perturbed realization is treated as a query, and its unperturbed parent must be recovered from the complete library of 500 reference reconstructions. Cosine similarity, Pearson correlation, cross-cosine similarity, and cross-correlation are evaluated separately before any combined score is considered. These four measures form two directly comparable pairs: fixed-grid correlation and angular similarity, and their rigid-lag counterparts. DTW and SFEC remain in the perturbation-response analysis but are not included in this targeted ranking experiment. We report Top-1, Top-5, Top-10, Top-20, reciprocal rank, and full-library correct-parent rank so that the role of each selector remains visible.

Complementarity is then evaluated with a convex score $H_{w}=\left(1-w\right)M_{\mathrm{base}}+wS_{J}$, where $M_{\mathrm{base}}$ is the selected waveform score mapped to [0,1] and the same global weight *w* is used for every query and every selector. Both terms are available for all 500 candidates, so the hybrid ranks the complete library without a candidate preselection step. The $S_{J}$ parameter configuration and *w* are selected on calibration references only. The selected settings are frozen before evaluation on held-out references, and all 50,000 queries are retained in both the standalone and combined retrieval analyses. This remains a controlled parent-spectrum recovery task; it does not substitute for blind identification with experimental replicates and a chemically curated near-neighbor library.

This setup remains a reference-preserving robustness experiment. Because each query is generated from a known reference, the measured outcome is the response of each metric to controlled degradation and structural modification. Retrieval is used here as an operational test of the metric cascade, not as a fully blind identification study.

### 2.5. Lorentzian Peak-Table Construction

Individual Raman bands are represented by the Lorentzian line shape

(16) $$L\left(x;x_{0},\sigma ,A\right)=\frac{A}{\pi }\frac{\sigma }{{\left(x-x_{0}\right)}^{2}+\sigma^{2}},$$

where $x_{0}$ is the band center, σ is the half-width at half maximum, and *A* is the integrated amplitude. The peak height is A/(πσ) and the FWHM is 2σ. A spectrum containing *K* bands is reconstructed as $\hat{y}\left(x\right)=b+\sum_{j=1}^{K}L\left(x;x_{0j},\sigma_{j},A_{j}\right)$. The benchmark assumes baseline-corrected inputs and uses b=0 for the spectra entering the similarity analysis.

Savitzky–Golay smoothing may be applied before peak detection when high-frequency noise obscures local maxima [41]. The smoothed signal is used only to locate candidate bands and estimate their initial widths; parameter optimization is performed against the original spectrum. Candidate centers are selected using minimum-prominence and minimum-distance criteria, and initial FWHM values are estimated from the widths measured at a specified relative height.

The number of components may be fixed from prior information or selected automatically. In the latter case, candidate decompositions of increasing complexity are fitted and compared using the Bayesian Information Criterion. Centers, widths, and amplitudes are refined by bounded least squares. Positive-amplitude constraints and prespecified center and FWHM bounds restrict the search to interpretable parameter ranges; they do not guarantee a unique physical decomposition.

This automated decomposition provides a reproducible preliminary peak table for the benchmark. It is not part of the mathematical definition of $S_{J}$, which operates on centers, relative heights, and width descriptors after a peak table has been supplied. A user may instead provide an expert-curated or externally fitted table, or inspect and adjust the preliminary table through the online interface. Supplementary Sections S1.1 and S1.9 give the complete fitting procedure, an independent comparison with Fityk [29], and the editable-fit workflow.

### 2.6. Perturbations and Artifacts Applied to Spectra

In this section, we define perturbations that emulate common sources of Raman variability and implement them as *peak-table augmentations* plus controlled additive noise. This implementation consists of constructing each perturbed spectrum by modifying peak parameters and re-synthesizing the spectrum as a sum of Lorentzian components (Supplementary Section S1.1). The perturbation procedure is summarized in Algorithm 2 and is detailed in the following sections. Within the workflow, the perturbation model is the stress-test layer. Its distributions define a controlled, source-agnostic test rather than a fitted model of one instrument, database, or sample-preparation protocol. The axes correspond to variability reported in Raman practice: intensity-ratio changes, Raman-shift drift, linewidth variation, peak loss, spurious features, and signal-to-noise degradation [12,33,34,35,42]. CaPSim provides a related SERS-recognition example in which small peak shifts, residual backgrounds, low-intensity noisy features, and substrate-dependent variability complicate matching to Raman libraries [28].
**Algorithm 2** Peak-based spectral perturbation generation**Require:** Initial peak table $P_{s,0}$ with tuples $\left(x_{0j},\sigma_{j},A_{j}\right)$
**Require:** Perturbation parameters Π and spectral grid *x*
**Ensure:** Perturbed peak table $P_{s,r}$ and spectrum $y_{s,r}\left(x\right)$
  1:Generate smooth random fields $f_{\mathrm{shift}},f_{\mathrm{gain}},f_{\mathrm{fwhm}}$ on u∈[0,1]  2:**for** each peak *j* **do**  3:    Compute correlated shift $\Delta x_{j}$, gain $g_{j}$, and broadening $b_{j}$  4:    Update $x_{0j}^{\prime }$, $\sigma_{j}^{\prime }$, and $A_{j}^{\prime }$  5:**end for**  6:Drop peaks according to height-weighted probabilities  7:Mutate retained peaks with probability $p_{\mathrm{mut}}$  8:Inject additional weak peaks under exclusion constraints  9:Reconstruct ${\hat{y}}_{s,r}\left(x\right)$ from the perturbed peak set 10:Add Gaussian noise to reach the target SNR 11:**return**$P_{s,r}$, $y_{s,r}\left(x\right)$

#### 2.6.1. Peak-Table Representation

Each spectrum is represented by a peak table obtained through Lorentzian fitting (Supplementary Section S1.1):

(17) $$P={\left\{\left(x_{0j},\sigma_{j},A_{j}\right)\right\}}_{j=1}^{K},$$

where $x_{0j}$ is the center (cm^−1^), $\sigma_{j}$ is the Lorentzian scale parameter (half-width at half-maximum), and $A_{j}$ is the Lorentzian amplitude. The FWHM is $\Gamma_{j}=2\sigma_{j}$. In this parameterization, the peak height is

(18) $$H_{j}=\frac{A_{j}}{\pi \sigma_{j}}.$$

This representation makes it natural to implement controlled modifications in (i) peak position, (ii) intensity, and (iii) linewidth.

#### 2.6.2. Smooth Random Fields for Shift, Gain, and Broadening

A central design choice of the proposed perturbation framework is that changes in peak position, intensity, and width are *not* generated independently for each band. Instead, they are produced by smooth random fields defined along the spectral axis. This reflects realistic experimental conditions, where calibration drift, optical misalignment, or preprocessing artifacts tend to induce *correlated* distortions across neighboring spectral features rather than isolated, uncorrelated changes.

To this end, we introduce a normalized coordinate u∈[0,1] spanning the spectral range (either continuously along the grid or evaluated at peak locations). For each perturbation type, we construct a smooth scalar field

(19) P(u)∈[−1,1],

obtained from low-degree random polynomials (degree 4 in this work) whose values are centered, smoothly varying, and constrained to vanish at the boundaries. This construction ensures global smoothness, avoids edge instabilities, and yields perturbations with both positive and negative regions distributed over the spectrum.

Three independent smooth fields are generated:

(20) $$f_{\mathrm{shift}}\left(u\right),\;f_{\mathrm{gain}}\left(u\right),\;f_{\mathrm{fwhm}}\left(u\right),$$

which are evaluated at the normalized peak locations $u_{0j}$ to produce correlated, per-peak perturbation factors.

##### (P1) Raman-Shift Field

Small calibration or alignment drifts typically manifest as correlated shifts in peak positions [43]. Using the smooth shift field, each peak center is displaced by a signed amount

(21) $$\Delta x_{j}=\delta_{\max}\;f_{\mathrm{shift}}\left(u_{0j}\right),\;f_{\mathrm{shift}}\left(u_{0j}\right)\in \left[-1,1\right],$$

so that peak positions may shift in either direction along the Raman-shift axis. The perturbed center is then given by

(22) $$x_{0j}^{\prime }=x_{0j}+\Delta x_{j}.$$

In this study, $\delta_{\max}=30\;{\mathrm{cm}}^{-1}$. Here, *symmetric* means that shifts are generated around zero (no enforced global sign), so positive and negative local displacements are both allowed across the spectrum, rather than imposing a one-direction drift.

Raman-shift displacement can arise from instrumental calibration and registration, changes in phase or chemical environment, and Doppler-related effects in applicable acquisition geometries. The smooth shift field represents the resulting displacement phenomenologically; application-specific attribution of the shift requires the corresponding acquisition metadata and a dedicated calibration study.

Such small yet systematic band displacements are not merely instrumental artifacts but are also commonly observed when the physical or chemical environment of a compound changes. In biological samples and diluted solutions, Raman bands may undergo subtle shifts of only a few cm^−1^ while preserving the overall spectral fingerprint. For example, comparative studies of amino acids in crystalline form and in aqueous solution report measurable Raman-band shifts attributed to solvation and hydrogen-bond interactions that perturb intramolecular force constants [44]. More generally, differences between solid-state and solution-phase spectra are well documented and arise from changes in intermolecular coupling, crystal-field effects, and the disappearance of Davydov splitting upon dissolution [45]. Consequently, spectra recorded under varying concentration, temperature, or matrix conditions often exhibit small but physically meaningful misalignments that can challenge strictly pointwise similarity metrics.

##### (P2) Intensity Gain Field

Global variations in laser power, collection efficiency, or normalization imperfections are modeled via a smooth multiplicative field [14,42]:

(23) $$g_{j}=1+\lambda_{I}\;f_{\mathrm{gain}}\left(u_{0j}\right),$$

where $\lambda_{I}\in \left(0,1\right)$ controls the maximum relative intensity variation. Gain is applied at the amplitude level,

(24) $$A_{j}^{\prime }=A_{j}\;g_{j},$$

ensuring physically consistent scaling of peak height and area. Here, $\lambda_{I}=0.3$, which yields bounded gain factors in approximately [0.7,1.3].

##### (P3) Broadening (FWHM) Field

Changes in instrumental resolution, focus, or local sample environment often lead to correlated linewidth variations [34,46,47]. We model this through a smooth broadening factor

(25) $$b_{j}=\max\left\{1+\lambda_{F}\;f_{\mathrm{fwhm}}\left(u_{0j}\right),\;b_{\min}\right\},$$

where $\lambda_{F}$ controls the strength of the effect and $b_{\min}>0$ prevents degenerate widths. The perturbed widths are

(26) $$\sigma_{j}^{\prime }=\sigma_{j}\;b_{j},\;\Gamma_{j}^{\prime }=2\sigma_{j}^{\prime }.$$

The values used here are $\lambda_{F}=0.3$ and $b_{\min}=0.1$.

#### 2.6.3. Discrete Peak-Level Events

While smooth fields capture correlated instrumental drift, real spectra also differ through *discrete structural events* such as peak disappearance, local spectral changes, or the appearance of spurious bands. These effects are modeled explicitly by the following stochastic mechanisms.

##### (P4) Peak Dropping (Disappearance)

Each original peak may be removed with a probability inversely related to its normalized height. Let

(27) $$h_{j}=\frac{H_{j}}{H_{\max}}\in \left[0,1\right]$$

denote the relative prominence of peak *j*. The drop probability is defined as

(28) $$p_{j}^{\mathrm{drop}}=p_{\max}^{\mathrm{drop}}\left(1-h_{j}\right),$$

so that weak peaks are more likely to disappear, reflecting realistic detection thresholds in low-SNR or strongly perturbed conditions [35,48]. To avoid degenerate realizations, at least one peak (typically the strongest) is always retained. We set $p_{\max}^{\mathrm{drop}}=0.04$.

##### (P5) Peak Mutation (Local Modification)

To model local spectral distortions, retained peaks may generate mutated offspring with probability $p_{\mathrm{mut}}$ [49]. A mutated peak is created by perturbing position, height, and width:

(29) $$x_{0}^{\mathrm{child}}=x_{0}+\Delta x,\;\Delta x∼U\left(-\Delta x_{\max},\Delta x_{\max}\right),$$

(30) $$H^{\mathrm{child}}=H\;\rho_{H},\;\rho_{H}∼U\left(\rho_{H}^{\min},\rho_{H}^{\max}\right),$$

(31) $$\Gamma^{\mathrm{child}}=\Gamma \;\rho_{\Gamma },\;\rho_{\Gamma }∼U\left(\rho_{\Gamma }^{\min},\rho_{\Gamma }^{\max}\right).$$

This mechanism introduces localized spectral changes while preserving the Lorentzian line-shape model. The values used here are $p_{\mathrm{mut}}=0.04$, $\Delta x_{\max}=15\;{\mathrm{cm}}^{-1}$, $\left(\rho_{H}^{\min},\rho_{H}^{\max}\right)=\left(0.2,0.7\right)$, and $\left(\rho_{\Gamma }^{\min},\rho_{\Gamma }^{\max}\right)=\left(0.8,1.2\right)$.

##### (P6) Peak Injection (Spurious Bands)

To emulate contamination, noise-induced features, or secondary phases [50], we inject a limited number of new weak peaks. Each injected center is sampled uniformly over the spectral range, but must satisfy an exclusion distance to avoid unrealistic peak overlap:

(32) $$|x_{0}^{\mathrm{inj}}-x_{0j}|\;>d_{\mathrm{excl}}\;\forall j,$$

Injected peak heights and widths are defined relative to the original spectrum:

(33) $$H^{\mathrm{inj}}=\alpha \;H_{\max},\;\alpha ∼U\left(\alpha_{\min},\alpha_{\max}\right),$$

(34) $$\Gamma^{\mathrm{inj}}=\eta \;\Gamma_{\mathrm{med}},\;\eta ∼U\left(\eta_{\min},\eta_{\max}\right).$$

The maximum number of injected peaks per realization is 2, with $d_{\mathrm{excl}}=25\;{\mathrm{cm}}^{-1}$, $\left(\alpha_{\min},\alpha_{\max}\right)=\left(0.05,0.15\right)$, and $\left(\eta_{\min},\eta_{\max}\right)=\left(0.8,1.2\right)$.

#### 2.6.4. Reconstruction and Gaussian Noise with Controlled SNR

After applying smooth fields and discrete peak-level events, a final perturbed peak set $P_{s,r}$ is obtained. The corresponding spectrum is reconstructed as

(35) $${\hat{y}}_{s,r}\left(x\right)=\sum_{j\in P_{s,r}}L\left(x;x_{0j}^{\prime },\sigma_{j}^{\prime },A_{j}^{\prime }\right)+b,$$

where *b* denotes the baseline, which is set to zero because the spectra are assumed to have been baseline-corrected before entering the benchmark.

Finally, additive Gaussian noise is introduced to achieve a prescribed signal-to-noise ratio:

(36) $$y_{s,r}\left(x\right)={\hat{y}}_{s,r}\left(x\right)+\varepsilon \left(x\right),\;\varepsilon \left(x\right)∼N\left(0,\sigma_{N}^{2}\right),$$

with $\sigma_{N}=\sigma_{\mathrm{signal}}/\mathrm{SNR}$, where $\sigma_{\mathrm{signal}}$ is the empirical standard deviation of the reconstructed signal. Gaussian white noise has been used in simulated Raman denoising studies, and Gaussian readout noise is one recognized contribution to spectroscopic noise [51,52]. Here it provides a reproducible SNR axis; it is not presented as a universal detector-noise model. For the benchmark runs reported here, SNR is sampled as integers in [1,25], covering low- to moderately high-quality acquisition conditions.

After noise is added, the continuous-query spectrum is fitted independently to obtain the peak table used by $S_{J}$. The hidden perturbed table that generated the continuous spectrum is retained only as simulation provenance and is not substituted for the noisy-query fit.

For each perturbed spectrum, summary statistics are recorded, including extrema and averages of Δx, relative intensity change, and FWHM variation, as well as the numbers of dropped, mutated, and injected peaks.

#### 2.6.5. Effects of These Perturbations on Similarity Metrics

The proposed perturbations are designed to probe complementary sensitivity and invariance properties of spectral similarity measures under controlled but realistic deviations. Each class of perturbation emphasizes a distinct aspect of spectral agreement that may be relevant depending on the application context:

- **Shift.** Correlated Raman-shift offsets test the sensitivity of similarity measures to small and local misalignments in peak position, as may arise from calibration drift or preprocessing variability.
- **Gain.** Smooth intensity scaling evaluates how similarity measures respond to global or slowly varying changes in signal amplitude, such as those caused by variations in excitation power or collection efficiency.
- **Broadening.** Linewidth perturbations assess the impact of changes in spectral resolution or peak sharpness, which modify local gradients while preserving overall band structure.
- **Dropping, injection, and mutation.** Discrete peak-level events introduce structural differences between spectra by altering the number, location, or shape of individual bands, thereby testing how similarity measures account for partial correspondence or missing information.
- **Noise and SNR.** Additive noise probes robustness under reduced measurement quality, particularly in regimes where weak spectral features approach the detection limit.

Together, these perturbations define a controlled stress test across diverse spectral distortions. Their ranges are prescribed analysis axes rather than empirical estimates of the frequency or joint distribution of effects in a particular instrument or sample population. The observed behaviors are discussed quantitatively in Section 3 and interpreted in the context of prior studies on spectral artifact sensitivity [12,33].

**Figure 2:**
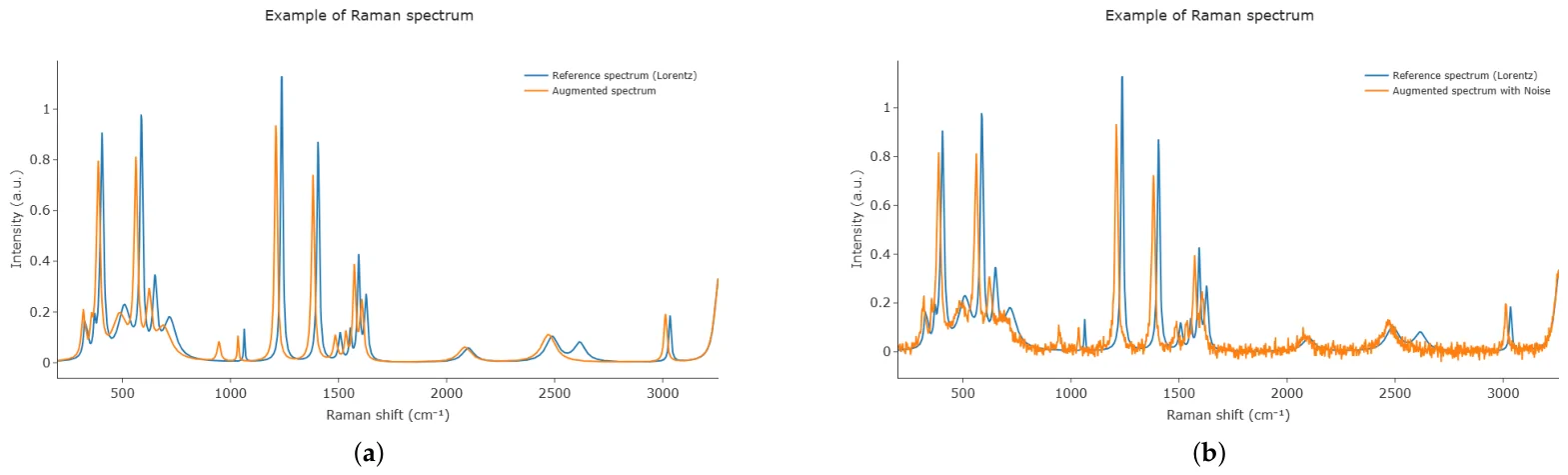
Illustrative example of the spectral perturbation model used in this work. The reference spectrum is reconstructed from a fitted peak table, then modified through smooth correlated fields (shift, gain, and broadening) and discrete peak events (dropping, mutation, and injection). Noise is added in a final step to emulate realistic measurement conditions. (**a**) Reference spectrum reconstructed from Lorentzian peaks (blue) and perturbed spectrum after peak-level modifications (orange), prior to noise injection. (**b**) Same realization after additive Gaussian noise at a controlled signal-to-noise ratio (SNR).

Figure 2 illustrates a representative realization of the spectral perturbation pipeline used throughout this study. In Figure 2a, the blue curve corresponds to the reference spectrum reconstructed from its Lorentzian peak table, while the orange curve shows the perturbed spectrum after applying peak-level modifications but before noise injection. Several effects discussed in Section 2.6 are visible simultaneously: correlated shifts in peak positions along the Raman-shift axis (P1), changes in relative peak intensities (P2), and peak broadening or narrowing through FWHM scaling (P3). In addition, discrete structural events can be observed, including the disappearance of a weak band, the appearance of a new spurious peak (P4, P6).

Figure 2b shows the same realization after the addition of Gaussian noise at a controlled signal-to-noise ratio. While the overall band structure remains recognizable, noise partially obscures weaker peaks and further degrades pointwise alignment. This example provides an intuitive visualization of how the synthetic perturbations translate into concrete spectral differences, complementing the statistical robustness analyses presented in the following sections.

### 2.7. $S_{J}$ Parameter Sensitivity, Calibration, and Freezing

The complete configuration $\theta_{S_{J}}$ contains the Hungarian weights and soft scales, the maximum center-displacement gate, the orphan costs, the match-quality scales for center, relative height, and FWHM, the orphan-severity scale, and the peak-weighting rule. These quantities define how deviations are graded and must therefore be reported as part of the score definition. In the present calibration, only the four scoring scales $\left(\tau_{x},\tau_{y},\tau_{F},\tau_{y}^{\mathrm{non}}\right)$ are varied; the assignment parameters, gate, orphan costs, and uniform peak weighting are fixed at the documented benchmark values. The formulation admits optional peak weights, but diagnostic-band and region-specific weighting strategies are not evaluated in this study.

We first evaluate prespecified one-at-a-time and factorial grids for these four tolerances on the calibration references. Selection uses the score response to a perturbation-burden index that combines the ranked magnitudes of the continuous and discrete perturbations and inverse SNR. The criterion rewards a monotonic decrease in $S_{J}$ with increasing burden, retains score dispersion, and penalizes saturation near zero or one. Library retrieval is not part of this parameter-selection objective. The selected setting is combined with the fixed assignment configuration for all subsequent analyses, and held-out identities do not influence this choice.

Peak weighting is already configurable in the metric and API, but the benchmark uses uniform weighting throughout. The same rule is therefore applied to every perturbation, spectrum, and selector. Future application-specific studies may evaluate prespecified non-uniform weights, including weights motivated by diagnostic bands or spectral regions; no such weighting analysis is part of the present study.

### 2.8. Assessment of Lorentzian Goodness of Fit

The agreement between each noisy-query spectrum and its multi-Lorentzian reconstruction is quantified by the coefficient of determination, $R^{2}$. The distribution is summarized by its mean, median, standard deviation, and empirical quantiles, and its dependence on noise is examined across five SNR intervals. These summaries describe the numerical agreement of the automatic decomposition with the query spectrum and are not used to define an acceptable-fit threshold. All 50,000 comparisons remain in the subsequent analyses. In applied use, the fitted table can be inspected and edited through the API or replaced with a table obtained by another method before evaluating $S_{J}$.

### 2.9. Retrieval Outcomes, Difficult Cases, and Uncertainty

The retrieval analysis reports cosine similarity, Pearson correlation, cross-cosine similarity, cross-correlation, and standalone $S_{J}$ before evaluating any hybrid score. Top-*k* recovery is the proportion of queries for which the known parent occurs within the first *k* ranked references. Additional summaries are the correct-parent rank $r_{q}$ and mean reciprocal rank, $\mathrm{MRR}=Q^{-1}\sum_{q=1}^{Q}r_{q}^{-1}$, for *Q* queries. For each of the 50,000 queries, all 500 references are scored by $S_{J}$, yielding 25,000,000 query–reference comparisons. The hybrid analysis evaluates a prespecified grid of *w* in $H_{w}=\left(1-w\right)M_{\mathrm{base}}+wS_{J}$ across the complete library. Candidate values from 0 to 1 in increments of 0.05 are assessed with the same value required for all four waveform selectors. Eligible weights must be non-inferior in calibration MRR for every selector; the prespecified rule then maximizes the minimum MRR improvement across selectors, followed by the mean improvement, and selects the smaller value when candidates differ by less than $10^{-4}$ in MRR. The resulting global value is applied without selector-specific or query-specific retuning to held-out references.

Difficult cases are classified by prespecified full-library rank changes: the hybrid rank of the correct parent is recorded as improved, worsened, or unchanged relative to the waveform selector, with promotion to Top-1 recorded separately. These classifications describe how the waveform and peak-level terms affect the correct-parent rank; they are not used to select parameters. Case-level records are retained in the public archive. Because every reference receives both scores, no query is excluded by candidate availability. Uncertainty is estimated by resampling reference identities with replacement so that the 100 related perturbations of a reference remain together. The same block-bootstrap principle is used for principal metric, perturbation, and retrieval summaries.

To support practical use, the accompanying application programming interface (API) [36] exposes both the full $S_{J}$ calculation and its constituent operations. It can compute waveform metrics, propose a multi-Lorentzian peak table, run Hungarian matching on supplied peak tables, and return match lists, orphan counts, and per-peak discrepancy terms. The automatic fit is not presented as an immutable result. In the interactive workflow, a user can inspect the proposal, change peak centers, heights, or widths, add or remove components, and recompute the fit and $S_{J}$. A user may also bypass the Lorentzian proposal and supply a peak table obtained elsewhere. This modular access supports a complementary workflow in which rapid ranking, alignment-aware evidence, and band-level structure remain distinguishable.

## 3. Results and Discussion

The results follow the sequence of the study design. We first quantify the agreement between each noisy spectrum and its automatic Lorentzian representation. The $S_{J}$ tolerances are then selected using only the 350 calibration references and fixed before analysis of the 150 held-out references. We next examine sensitivity to the controlled perturbations and the dependence among the evaluated measures. Finally, we report the individual full-library rankings, the complementary hybrid rankings obtained with one calibration-selected global weight, the associated uncertainty, and the observed rank changes in difficult cases.

### 3.1. Lorentzian Reconstruction Quality

**Figure 3:**
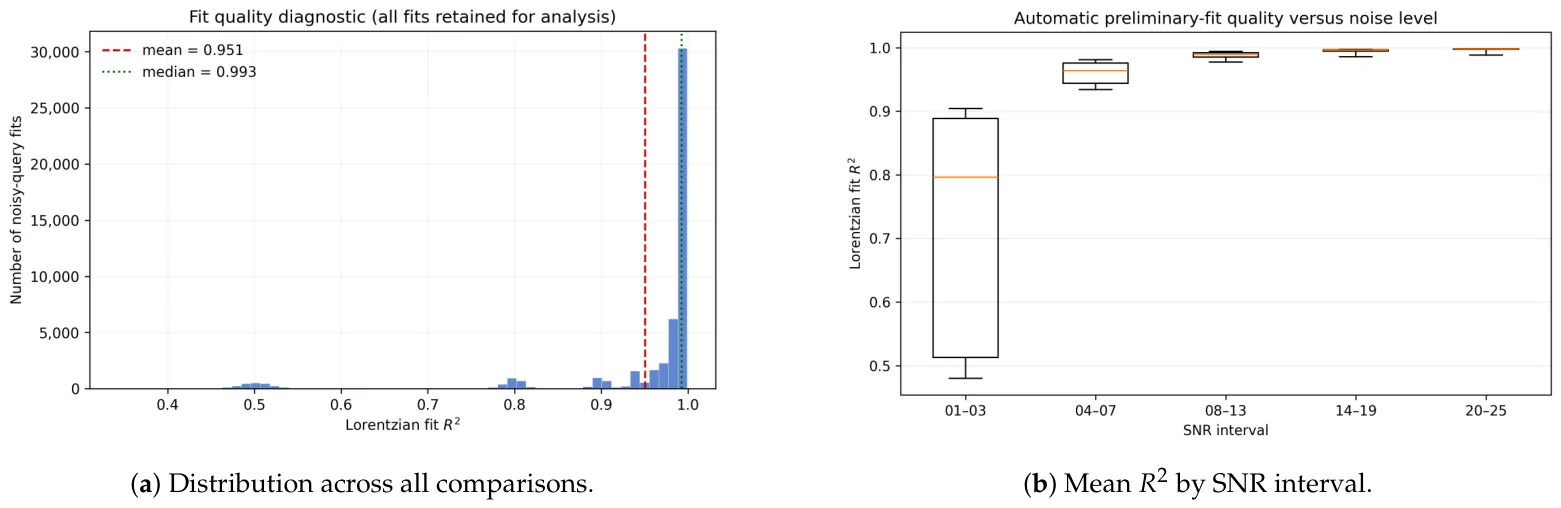
Goodness of fit of the automatic multi-Lorentzian representation. (**a**) Distribution of $R^{2}$ for the 50,000 perturbed spectra. (**b**) Mean $R^{2}$ within the prespecified SNR intervals.

Across the 50,000 perturbed spectra, the Lorentzian reconstructions had a mean $R^{2}$ of 0.9506, a median of 0.9927, and a standard deviation of 0.1129. The 5th and 95th percentiles were 0.6683 and 0.9984, respectively (Figure 3). The lower tail was concentrated mainly among spectra with the most severe noise. $R^{2}$ is reported as a continuous description of reconstruction agreement, and no cutoff was used in the subsequent analyses.

The mean $R^{2}$ increased from 0.7209 at SNR 1–3 to 0.9611 at SNR 4–7, 0.9879 at SNR 8–13, 0.9943 at SNR 14–19, and 0.9963 at SNR 20–25. Thus, the automatic decomposition closely represented most spectra, while its limitations were concentrated at the most severe noise levels. These $R^{2}$ values are descriptive: no goodness-of-fit threshold was used to remove a spectrum or its $S_{J}$ value. This distinction is important because the Lorentzian procedure provides one route to the peak table, whereas $S_{J}$ can also be evaluated from a user-edited table or from peaks obtained by another fitting method.

### 3.2. Sensitivity and Selection of the $S_{J}$ Parameters

**Figure 4:**
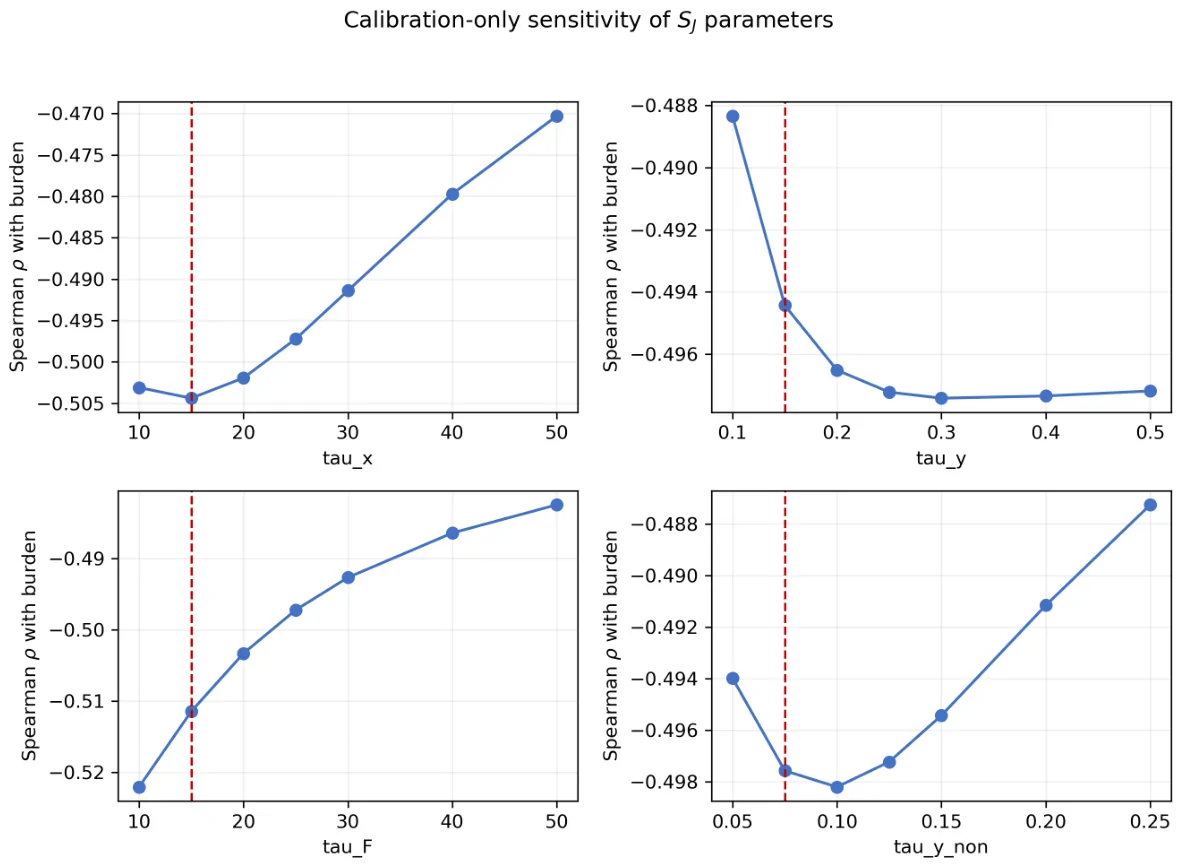
Calibration of the $S_{J}$ scoring tolerances. The curves show the effect of varying the center, relative-height, full width at half maximum (FWHM), and unmatched-peak tolerances on the calibration references. The selected operating point is indicated in the figure and was fixed before held-out evaluation.

Figure 4 shows how the score changed across the prespecified calibration grid. Larger tolerances made $S_{J}$ less sensitive to the corresponding deviation, as expected from the tolerance-controlled penalty; however, excessively permissive settings compressed the response and reduced its ability to distinguish perturbation burden. The selected tolerances were $\tau_{x}=15\;{\mathrm{cm}}^{-1}$, $\tau_{y}=0.15$, $\tau_{F}=15\;{\mathrm{cm}}^{-1}$, and $\tau_{y}^{\mathrm{non}}=0.075$ (Table 3). The assignment stage retained its fixed center and height weights, matching tolerances, and center-displacement gate. Uniform peak weighting was used throughout.

At the selected setting, the Spearman association between $S_{J}$ and the composite perturbation burden was −0.522 in the calibration set and −0.489 in the held-out set. The similar direction and magnitude in the two groups indicate that the selected response was not confined to the references used for calibration. Across the complete benchmark, the resulting $S_{J}$ values had a mean of 0.6464 and a median of 0.6577. These absolute values should be interpreted under the stated tolerances; they are not universal acceptance thresholds. The perturbation ranges were not restricted to values below those tolerances. In particular, the smooth center displacement reached $30\;{\mathrm{cm}}^{-1}$, twice the selected scoring scale $\tau_{x}=15\;{\mathrm{cm}}^{-1}$. Because each τ is the half-penalty scale of a smooth sigmoid, a deviation equal to τ already receives a penalty of 0.5, while larger deviations remain admissible and are penalized progressively. The tolerances therefore control the response of the score rather than defining a favorable or acceptable perturbation range.

### 3.3. Sensitivity to Controlled Perturbations

**Figure 5:**
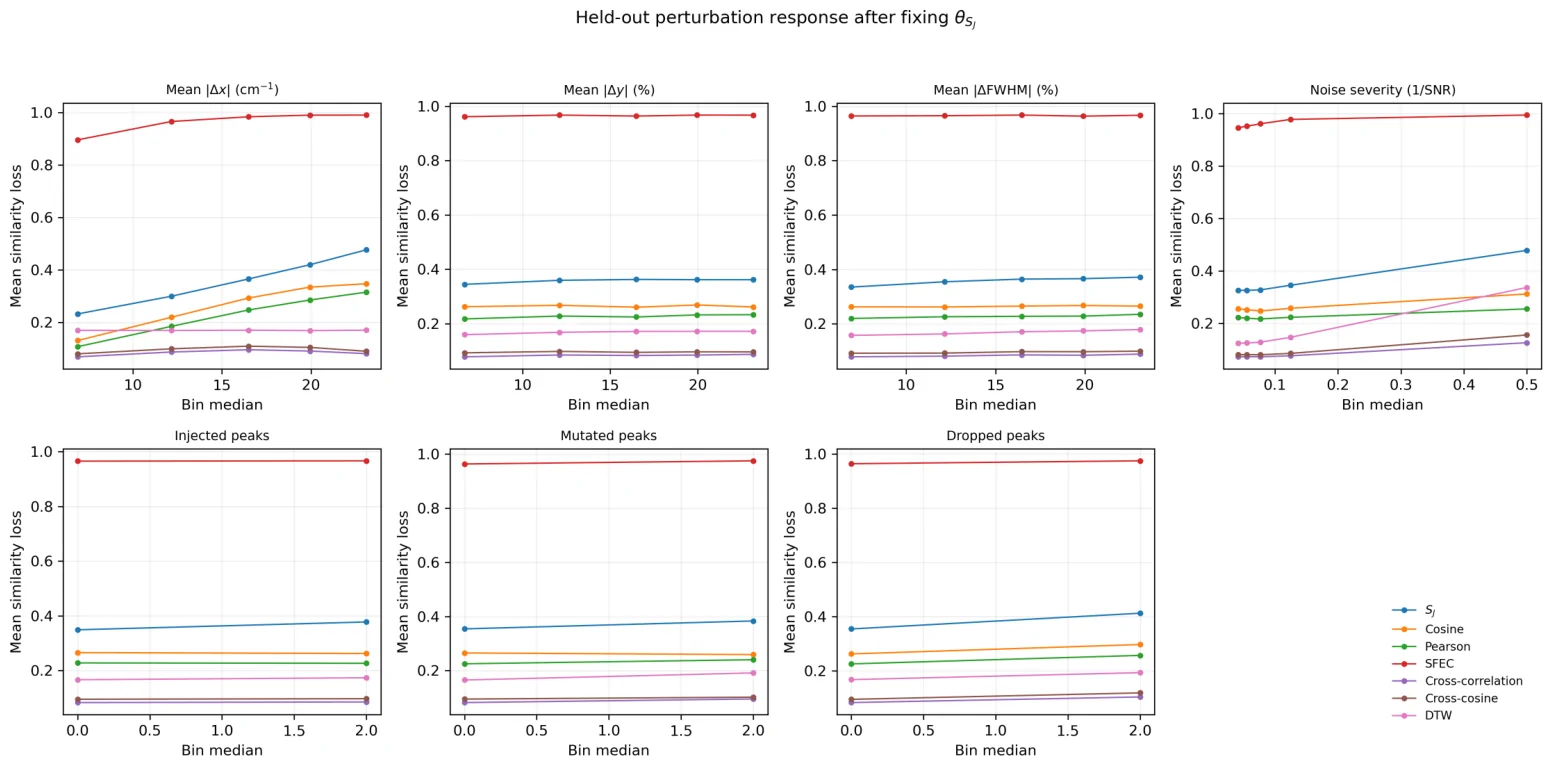
Held-out similarity response across the magnitudes of the continuous and discrete perturbations. Curves summarize observed metric changes within the mixed-perturbation design; shaded intervals are obtained by resampling reference identities.

**Figure 6:**
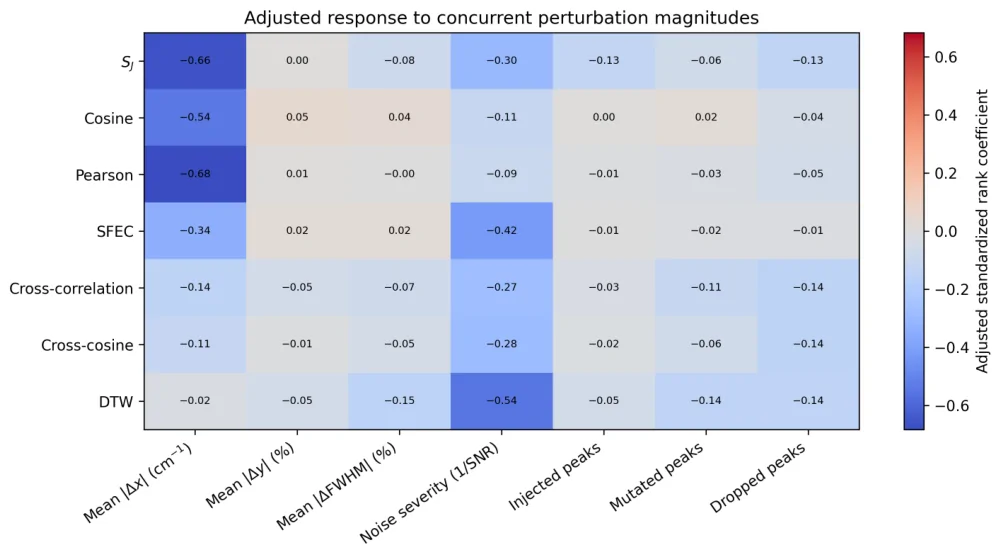
Adjusted rank associations between perturbation descriptors and similarity measures in the held-out set. Variables were rank-transformed, and the association with each descriptor was calculated after accounting for the remaining descriptors. The estimates are descriptive of the mixed benchmark and are not interpreted as causal effects.

The binned response curves in Figure 5 and the adjusted rank associations in Figure 6 distinguish the perturbations emphasized by each metric. For $S_{J}$, the largest held-out change between the lowest- and highest-burden bins occurred for peak-center shift: the median loss was 0.2448, with a 95% confidence interval (CI) of 0.2348–0.2541. Noise was the second largest continuous effect, with a loss of 0.1524 (95% CI, 0.1440–0.1616). The losses associated with FWHM and gain were smaller, at 0.0364 (95% CI, 0.0273–0.0459) and 0.0171 (95% CI, 0.0076–0.0260), respectively.

The explicit peak events produced $S_{J}$ losses of 0.0581 for dropped peaks (95% CI, 0.0486–0.0687), 0.0292 for mutated peaks (95% CI, 0.0208–0.0376), and 0.0286 for injected peaks (95% CI, 0.0235–0.0340). These effects are modest relative to the largest shift and noise contrasts because each query combines several perturbations and because the soft matching rule can preserve partial agreement. Nevertheless, all three intervals excluded zero, which is consistent with the intended orphan and match-quality terms.

The comparison metrics followed different profiles. At the extreme shift bin, Pearson correlation and cosine similarity lost 0.2081 and 0.2171, whereas the corresponding losses for cross-correlation and cross-cosine similarity were only 0.0121 and 0.0099. Conversely, DTW was most affected by noise (loss 0.2123) and showed no clear extreme-bin shift loss (0.0006, with an interval spanning zero). SFEC showed intermediate losses for shift (0.0945) and noise (0.0480). These contrasts explain why no single metric can represent direct overlap, rigid alignment, elastic alignment, and band structure equally well.

### 3.4. Complementarity Among Similarity Measures

**Figure 7:**
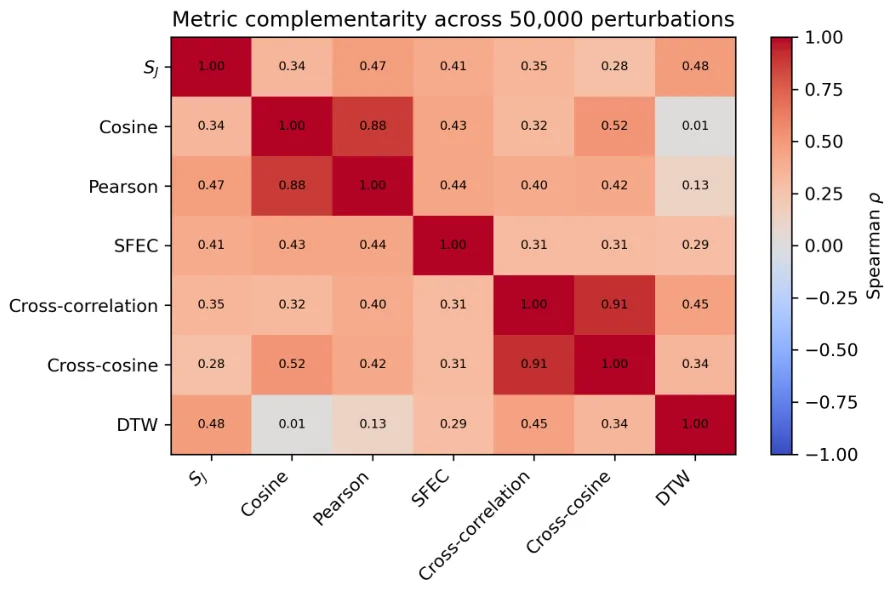
Spearman correlations among the evaluated similarity measures across the complete perturbation benchmark. Lower correlations identify metrics that retain more distinct information; they do not by themselves establish better performance.

The correlation structure in Figure 7 shows that $S_{J}$ did not duplicate any waveform metric. Its Spearman correlation ranged from 0.276 with cross-cosine similarity to 0.480 with DTW similarity; the corresponding values were 0.341 for cosine similarity, 0.474 for Pearson correlation, 0.408 for SFEC, and 0.351 for cross-correlation. By comparison, cosine similarity and Pearson correlation were strongly correlated ($\rho_{s}=0.877$), as were cross-correlation and cross-cosine similarity ($\rho_{s}=0.908$).

This separation is consistent with the perturbation responses and with the metric definitions. Pearson correlation and cosine similarity compare intensities on a common grid; their cross variants admit a rigid lag; DTW permits local elastic alignment; SFEC emphasizes first-order spectral change; and $S_{J}$ evaluates explicit band correspondence, including unmatched bands. Consequently, disagreement between two scores can be informative. A high cross metric with a lower pointwise score suggests recoverable misregistration, whereas a high waveform score with a lower $S_{J}$ points to disagreement in the band representation or to missing, additional, or substantially altered peaks. The results therefore support a complementary interpretation rather than a universal ranking of metrics.

### 3.5. Reference-Library Retrieval by the Individual Selectors

Library retrieval was evaluated on the 15,000 queries associated with the 150 held-out references. The two rigid-alignment selectors were strongest as standalone rankings: cross-correlation and cross-cosine similarity achieved Top-1 accuracies of 94.49% and 95.23%, respectively, compared with 41.66% for cosine similarity and 39.60% for Pearson correlation (Table 4). The result is consistent with the perturbation study because the library task includes center shifts that penalize fixed-grid comparisons. It does not make the alignment-aware metrics universally preferable: they answer whether a candidate becomes similar after an allowed lag, whereas the pointwise metrics retain stricter information about direct superposition.

### 3.6. Complementary Full-Library Ranking with a Single Global Weight

**Figure 8:**
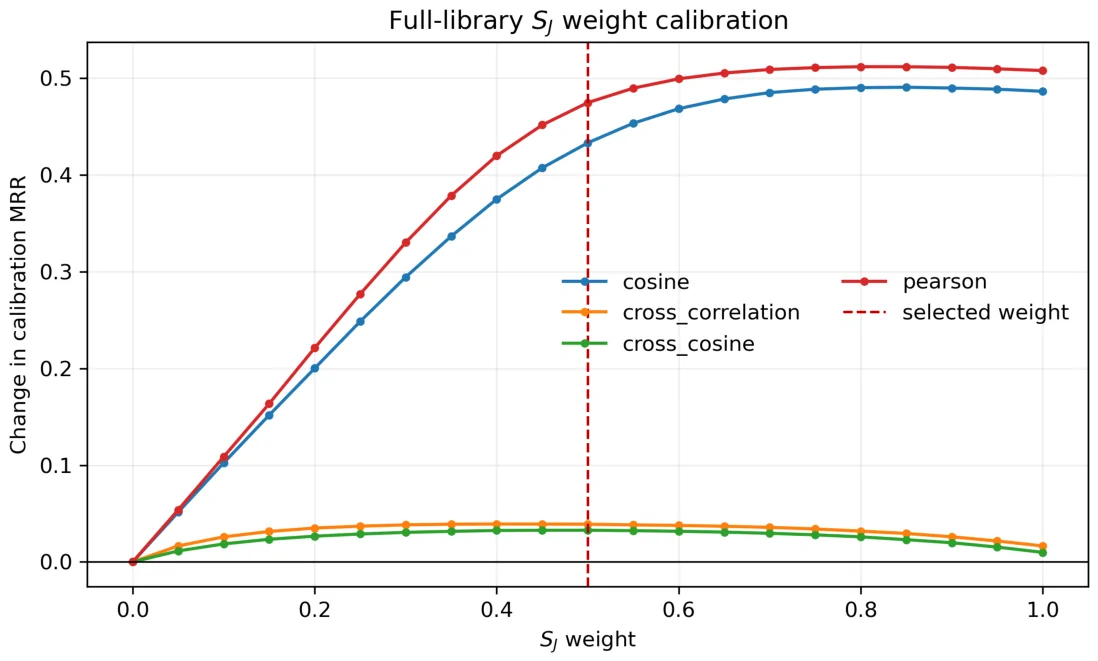
Calibration-set change in mean reciprocal rank as a function of the global $S_{J}$ weight. Every curve is based on a full 500-reference ranking. The vertical marker identifies the selected value w=0.50, which was applied unchanged to every selector and held-out query. At w=1, only $S_{J}$ determines the common full-library order.

Calibration selected w=0.50 in $H_{w}=\left(1-w\right)M_{\mathrm{base}}+wS_{J}$ using the single global rule defined in Section 2.9. This value was fixed before the held-out evaluation and was not retuned for any selector or query (Figure 8). Because both terms were calculated for every query–reference pair, the hybrid ranked all 500 references at every value of *w*; at w=1, all four curves therefore represent the same standalone $S_{J}$ ranking.

**Figure 9:**
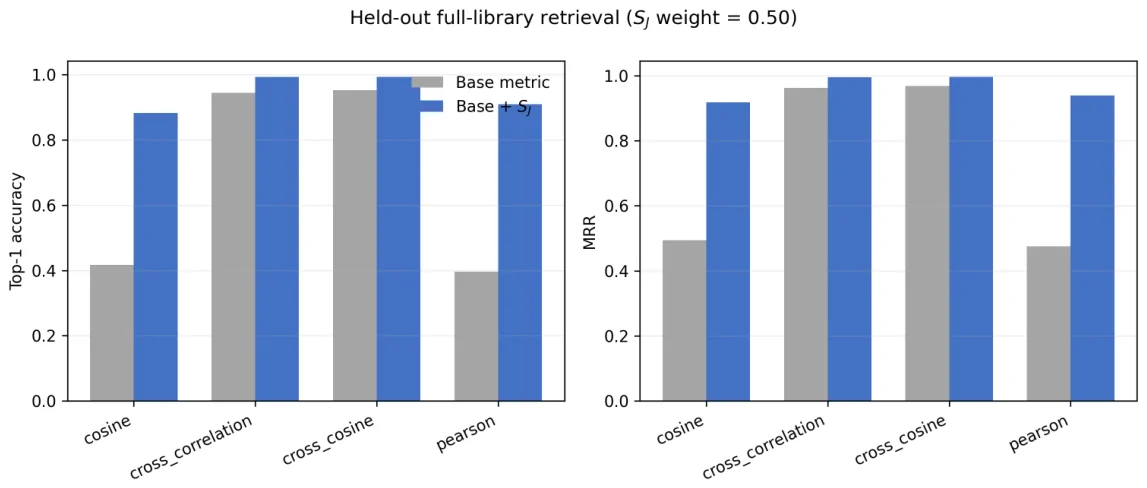
Held-out full-library parent-spectrum retrieval. The gray bars show the standalone waveform selectors and the blue bars show the corresponding hybrids with w=0.50. Both Top-1 accuracy and mean reciprocal rank use all 500 references for every query.

Standalone $S_{J}$ achieved a held-out Top-1 recovery of 96.03% and an MRR of 97.07% (Table 4). The hybrid improved all four waveform rankings (Table 4; Figure 9). Top-1 recovery increased from 41.66% to 88.28% for cosine similarity and from 39.60% to 90.94% for Pearson correlation. The already strong alignment-aware rankings also improved, from 94.49% to 99.30% for cross-correlation and from 95.23% to 99.33% for cross-cosine similarity. The larger gains for the fixed-grid measures reflect their vulnerability to the simulated shifts, whereas the smaller gains for the rigid-lag measures arise from their high standalone performance. All four paired Top-1 confidence intervals excluded zero.

### 3.7. Rank Changes and Difficult Cases

Because every query was compared with all 500 references, the rank-change analysis is not limited by a preliminary candidate list (Supplementary Section S1.6). The hybrid improved the correct-parent rank for 57.8% of cosine queries and 60.0% of Pearson queries, while worsening it for only 0.37% and 0.35%, respectively. Cross-correlation and cross-cosine began much closer to the performance ceiling; their ranks improved for 5.39% and 4.64% of queries and worsened for 0.50% and 0.42%.

The largest gains occurred for the two fixed-grid measures, whose standalone ranks are most affected by horizontal displacement. For the alignment-aware selectors, most queries were already correct at rank one, so the number of possible improvements was smaller; nevertheless, the hybrid improved their Top-1 and MRR values without selector-specific tuning. The prespecified difficult-case categories and archived case-level records permit inspection of the corresponding full-library rank changes and band-level matches; neither was used to select the global weight.

### 3.8. Scope and Interpretation

The reference-level bootstrap intervals account for the 100 related perturbations derived from each source spectrum; treating the 50,000 queries as independent would have understated uncertainty. The consistent held-out retrieval gains and the distinct perturbation profiles support the central interpretation of the study: the metrics are complementary because they preserve different forms of spectral evidence. $S_{J}$ adds explicit band correspondence and unmatched-band information, but it does not replace pointwise or alignment-aware measures.

Several boundaries remain. The benchmark assumes that baseline correction has already been performed and that a peak table is available. The automatic Lorentzian procedure is a reproducible preliminary fit, not a unique physical decomposition; it can be inspected, edited, or replaced in the accompanying API. The retrieval experiment recovers the known parent spectrum under controlled perturbations and should not be interpreted as blind chemical identification. The 500-reference library also does not represent every form of Raman data, particularly broad biological bands, SERS, hyper-Raman spectra, strong fluorescence backgrounds, or densely populated libraries of near-neighbor compounds. Validation of the framework in these settings remains future work and will require representative data, application-specific preprocessing and fitting choices, and independent calibration. The benchmark does not model grain-size effects, laser-heating dynamics, non-stationary scattering, Doppler shifts, or missing low-shift measurements as separate physical mechanisms. Its Raman-shift displacement is phenomenological, and interpretation below $200\;{\mathrm{cm}}^{-1}$ remains conditional on the measured range and instrument filters of the source spectrum. Future work may also examine prespecified non-uniform peak weights or additional peak descriptors; although $S_{J}$ already accepts peak weights, their application-specific calibration and validation were not studied here.

## 4. Computational Resources and Supplementary Materials

A web-based API accompanies this study to support online use of the implemented metric set. The FastAPI service (version 1.2.0) [36] provides documented endpoints for multi-metric comparison, standalone Lorentzian fitting, and standalone Hungarian peak matching, together with an interactive browser interface for fitting and inspecting article results. The automatic Lorentzian output is a preliminary proposal: the interactive interface allows the user to modify peak centers, heights, and widths, add or remove components, or replace the proposal with an external peak table before matching and $S_{J}$ evaluation. Initial access after a period of inactivity may be delayed while the hosting platform starts the service.

Supplementary Material S1 provides the extended fitting and peak-assignment details, the complete $S_{J}$ sensitivity outputs, an illustration of shift sensitivity, a concise illustrated API quick-start, explicit instructions for reviewing and modifying an automatic Lorentzian proposal, and the benchmark-archive description. Manual editing is an expert-inspection facility and is not used to alter the automated benchmark results. The supporting dataset is available through Zenodo [39].

## 5. Conclusions

This study establishes a controlled framework for comparing complementary Raman similarity measures under peak shift, gain, linewidth, discrete peak events, and noise. Its contributions are the integrated perturbation and evaluation pipeline, a calibrated peak-oriented similarity index, a 500-reference library-search task, and reference-level uncertainty estimates. The results show that the metrics answer different questions: pointwise measures quantify direct superposition, cross metrics tolerate a rigid offset, DTW permits elastic alignment, and $S_{J}$ evaluates explicit band correspondence and unmatched peaks.

The $S_{J}$ parameters were selected on 350 calibration references and fixed before evaluation on 150 held-out references. Full-library evaluation required 25,000,000 comparisons because each of the 50,000 queries was scored against all 500 references. Standalone $S_{J}$ achieved 96.03% held-out Top-1 recovery. With one global weight of 0.50, adding $S_{J}$ to each waveform selector improved held-out Top-1 parent recovery across the complete library. The increase ranged from 4.10 percentage points for cross-cosine similarity to 51.34 points for Pearson correlation, and every reference-level 95% confidence interval excluded zero. The common gain across selectors, together with the modest correlations between $S_{J}$ and the waveform metrics, supports complementarity rather than universal superiority.

The conclusions apply to baseline-treated spectra with identified peaks and to the perturbation ranges studied here. Lorentzian fitting is useful for generating an interpretable peak table, but it is not part of the mathematical definition of $S_{J}$ and need not be performed by the supplied routine. The online API therefore treats the automatic fit as an editable proposal and accepts externally obtained peak tables. Within these limits, the framework provides a reproducible way to combine library ranking with interpretable band-level evidence while keeping the role of each metric explicit. Although $S_{J}$ already supports alternative peak weights, evaluating region- or application-specific weighting strategies remains future work.

## Figures and Tables

**Table 1:** Relation between representative prior Raman-comparison families and the diagnostic role of the present benchmark.

Method Family	Typical Evidence Used	Main Practical Strength	Distinction in the Present Work
Correlation/HQI and mathematical spectral scores [18]	Full spectral vectors or transformed vectors	Efficient ranking and simple library-search scores	Used here as waveform evidence; structural support is assessed separately through peak correspondence
Segmental or voting-based HQI variants [7,9]	Windowed spectra or repeated local/class decisions	Reduces dependence on a single global score and can emphasize informative regions	Compared with a peak-oriented score that returns explicit matched and orphan peaks rather than segment scores alone
Raman-specific vector/windowed metrics [10]	Spectral neighborhoods, modified distance weights, and mixture-oriented intensity conditions	Encodes domain knowledge beyond generic pointwise scores for library search	Distinguished from $S_{J}$ because it does not return one-to-one peak assignments, orphan accounting, or per-band discrepancy terms
Characteristic-peak recognition strategies, including CaPSim and RIICP [24,28]	Characteristic peak regions or relative intensity relations between characteristic peaks	Supports Raman/SERS identification and false-positive filtering under nuisance variability	In the present pairwise robustness benchmark, explicit peak-table correspondence is used to interpret how structural agreement changes under controlled perturbations
Peak fitting and model selection [22,23,29]	Fitted centers, widths, amplitudes, and fit statistics	Makes spectral bands inspectable and parameterized	Peak tables are used as an inspectable layer for $S_{J}$, while the uncertainty of automatic peak recovery remains part of the interpretation
Artifact, preprocessing, and instrument-response studies [11,12,16,33,34,35]	Controlled or observed effects of noise, shifts, baseline, instrument response, and preprocessing choices	Shows why metric behavior depends on the artefact profile, acquisition conditions, and preprocessing choices	Motivates a mixed perturbation stress test with explicitly defined artefact axes

*Abbreviations:* HQI, hit quality index; CaPSim, Characteristic Peak Similarity; RIICP, Relative Intensity Information between Characteristic Peaks; SERS, surface-enhanced Raman scattering.

**Table 2:** Composition of the 500-reference collection and identity-level analysis partition. NASA denotes the National Aeronautics and Space Administration; RAMdb denotes the Raman Spectroscopic Database; OSU is retained as the source label used in the public collection.

Source	Total References	Calibration	Held-Out
NASA RAMdb	17	12	5
OpenSpecy-derived collection	17	13	4
OSU collection	15	9	6
Raman Open Database	55	39	16
RRUFF	145	109	36
RamanSPy	251	168	83
Total	500	350	150

**Table 3:** Configuration used for the final $S_{J}$ analyses.

Stage	Parameter	Value
Peak assignment	Center weight, $\alpha_{x}$	1.0
Peak assignment	Relative-height weight, $\alpha_{y}$	0.5
Peak assignment	Center tolerance, $\tau_{x}^{H}$	$20\;{\mathrm{cm}}^{-1}$
Peak assignment	Relative-height tolerance, $\tau_{y}^{H}$	0.10
Peak assignment	Maximum center displacement, $\Delta x_{\max}$	$50\;{\mathrm{cm}}^{-1}$
Match quality	Center tolerance, $\tau_{x}$	$15\;{\mathrm{cm}}^{-1}$
Match quality	Relative-height tolerance, $\tau_{y}$	0.15
Match quality	FWHM tolerance, $\tau_{F}$	$15\;{\mathrm{cm}}^{-1}$
Unmatched peaks	Relative-height tolerance, $\tau_{y}^{\mathrm{non}}$	0.075
Peak weighting	$\omega_{i}$	Uniform

**Table 4:** Held-out full-library retrieval for the standalone scores and the hybrids with w=0.50. Top-*k* and mean reciprocal rank (MRR) values are percentages. Confidence intervals (CI) refer to the paired Top-1 percentage-point change from the corresponding waveform selector and were obtained from 1000 bootstrap resamples of reference identities.

Selector	Ranking	Top-1	Top-5	Top-10	Top-20	MRR	Top-1 Change (95% CI)
Cosine	Standalone	41.660	57.080	63.960	71.873	49.412	–
	Hybrid	88.280	96.200	98.027	99.160	91.784	46.620 (44.266–48.960)
Pearson	Standalone	39.600	54.887	62.947	73.053	47.499	–
	Hybrid	90.940	97.620	98.740	99.460	93.914	51.340 (48.873–53.607)
Cross-correlation	Standalone	94.487	98.373	99.193	99.580	96.206	–
	Hybrid	99.300	99.893	99.953	99.980	99.568	4.813 (3.820–5.927)
Cross-cosine	Standalone	95.227	98.713	99.333	99.653	96.799	–
	Hybrid	99.327	99.940	99.973	99.980	99.606	4.100 (3.179–5.180)
$S_{J}$	Standalone	96.027	98.300	98.780	99.173	97.070	–

## Data Availability

The benchmark version supporting the present analyses is published on Zenodo [39]. It contains the reference and perturbed peak tables, the information required to reconstruct the noisy spectra, the complete metric results, the calibration and held-out assignments, the sensitivity and library-retrieval results, the bootstrap summaries, and source identifiers with original repository links and available experimental metadata. Supplementary Material S1 describes the data organization, the online FastAPI workflow, and the interactive Lorentzian-review interface.

## Supplementary Materials

The following supporting information can be downloaded at: https://www.mdpi.com/article/10.3390/mps9050148/s1, Supplementary Material S1 provides extended Lorentzian fitting and Hungarian-assignment details, the full $S_{J}$ calibration analysis, perturbation-stratified results, retrieval and bootstrap details, an illustrated FastAPI quick-start and editable-fit guide, and a description of the accompanying data collection. The data package contains source traceability and available experimental metadata, the 500 reference records, the reference and perturbed peak tables, the noisy-spectrum peak tables, the complete 50,000-comparison metric table, the calibration and held-out assignments, the perturbation and retrieval summaries, the bootstrap results, the figures, and the information needed to reproduce the simulated perturbations.

## Abbreviations

API	Application programming interface
APC	Article processing charge
BIC	Bayesian information criterion
CaPSim	Characteristic Peak Similarity
CI	Confidence interval
DTW	Dynamic time warping
FWHM	Full width at half maximum
HQI	Hit quality index
MRR	Mean reciprocal rank
NASA	National Aeronautics and Space Administration
RAMdb	Raman Spectroscopic Database
RIICP	Relative Intensity Information between Characteristic Peaks
SERS	Surface-enhanced Raman scattering
SFEC	Squared first-difference Euclidean cosine
SNR	Signal-to-noise ratio
