# Supplementary material for: Robust Comparison of Raman Spectra Under Various Perturbations: Benchmarking Common Similarity Metrics and a New Peak-Oriented Index
Source: Methods Protoc. 2026 Oct 9;9(5):148. doi: 10.3390/mps9050148 (PMC13707459; doi:10.3390/mps9050148)
Supplement: Supplementary file 1 [file mps-09-00148-s001.zip › mps-4513501-supplementary.pdf]

Supplementary Materials: Robust Comparison of Raman Spectra Under Various Perturbations: Benchmarking Common Similarity Metrics and a New Peak-Oriented Index

Adán Reyes-Reyes [1,2,3], Juan-Andrés Reyes-Reyes [1,2], Erick-Osvaldo Martínez-Ruíz [1,2], Alejandra Ortiz-Dosal [1,3], Ma. del Carmen Rodríguez-Aranda [1,2], Daniel-Carlos Reyes-Reyes [4], Stiven Villada-Gil [5], Luis Carlos Ortiz-Dosal [6], and Eleazar-Samuel Kolosovas-Machuca [1,2,*]

Scope

This document provides the implementation details and extended analyses supporting the Raman similarity study. Sections S1.1–S1.2 describe peak-table construction and Hungarian assignment. Sections S1.3–S1.6 specify the calibration split, the prespecified S_J tolerance search, Lorentzian reconstruction quality, perturbation-stratified analysis, reference-level bootstrap, full-library retrieval, and difficult-case rules. Section S1.7 illustrates the sensitivity of pointwise metrics to spectral shifts. Sections S1.8–S1.9 document the hosted application programming interface (API) [1], including a batch example and review of an automatically proposed Lorentzian fit. Sections S1.10–S1.11 describe the public data package [2] and the limits of interpretation.

The benchmark assumes that spectra have been treated for baseline and that a peak table is available or can be estimated. Baseline correction and peak extraction are preprocessing operations rather than parts of the definition of S_J. The automatic multi-Lorentzian procedure supplies a reproducible preliminary table, but a user may edit that proposal or provide a peak table obtained with another validated procedure. Goodness of fit is summarized by R^2 and is not used to exclude or downweight an S_J value.

S1.1. Lorentzian peak-table construction

Individual Raman bands are represented by the Lorentzian line shape

$$L(x; x_0, \sigma, A) = \frac{A}{\pi} \frac{\sigma}{(x - x_0)^2 + \sigma^2}, \tag{S1}$$

for which the peak height is $H = A/(\pi\sigma)$ and the full width at half maximum (FWHM) is $\Gamma = 2\sigma$. Given a peak table $\mathcal{P} = \{(x_{0j}, \sigma_j, A_j)\}_{j=1}^{K}$, the reconstructed spectrum is

$$\widehat{y}(x) = b + \sum_{j=1}^{K} L(x; x_{0j}, \sigma_j, A_j). \tag{S2}$$

The benchmark reconstructions use $b = 0$ because baseline-treated spectra are an input condition of the controlled study. Fluorescence, luminescence, and nonlinear backgrounds are not independently varied in the benchmark. The hosted API [1] can optionally apply one of its documented baseline-removal procedures to user-supplied spectra, but that convenience option does not change the assumptions or results of the article.

For automatic table construction, an optional Savitzky–Golay filter [3] is applied only to the signal used for initial peak detection. Parameter estimation is performed against the fitting trace defined by the selected fitting mode. Candidate centers are selected using minimum-prominence and minimum-distance criteria, and initial widths are estimated at a specified relative height. Shoulder detection and residual refinement provide additional candidates for congested or broad regions. The number of components may be fixed or

selected from an allowed range with the Bayesian Information Criterion (BIC). Centers, widths, and positive amplitudes are then refined by bounded least squares.

Algorithm S1 Automatic multi-Lorentzian peak-table construction

Require: Spectrum (x, y); optional smoothing; fixed K or candidate range; parameter bounds
Ensure: Peak table $\mathcal{P}$
1: Detect candidate centers on the original or optionally smoothed detection trace
2: Estimate initial widths and rank candidates by prominence
3: **if** K is fixed **then**
4: Fit the K-component model to the original spectrum
5: **else**
6: Fit each allowed candidate model to the original spectrum
7: Select the fitted model with minimum BIC
8: **end if**
9: **return** the selected peak table $\mathcal{P}$

Figure S1 compares one automatic decomposition with an independent fit obtained using *Fityk* [4]. It is a qualitative inspection example; the benchmark values are generated with one frozen automatic procedure. The coefficient of determination and recovered peak count describe the automatic proposal but do not determine whether a comparison is included in the analysis.

The definition of S_J does not require this particular fitting algorithm. It requires two consistently defined peak tables containing center, normalized height or intensity, and FWHM (or an equivalent width that can be converted to FWHM). A table may therefore come from expert curation, instrument software, a different line-shape model, or another validated fitting method. The origin of the table, normalization rule, spectral region, and units must be reported with the score. The online editor described in Section S1.9 supports review, modification, refitting, and export of the automatic proposal.

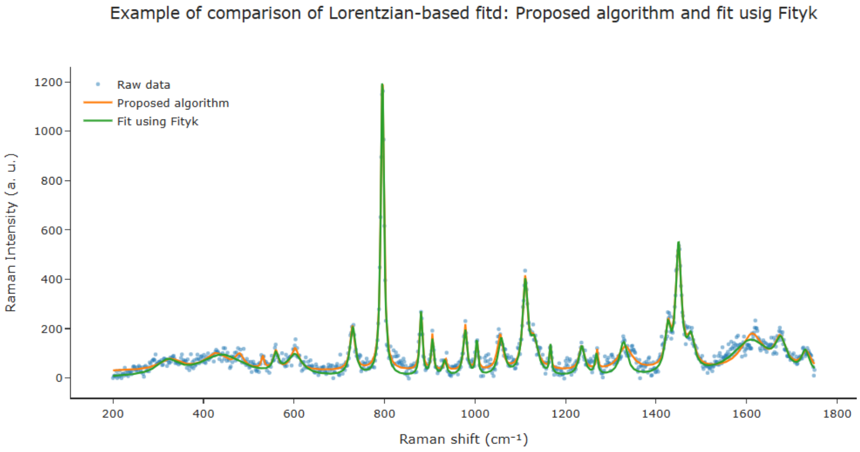

Figure S1. Example multi-Lorentzian decomposition. Blue: source spectrum; orange: automatic benchmark fit; green: independent *Fityk* fit [4].

S1.2. Hungarian assignment and orphan handling

Let $\mathcal{P}^* = \{(x_i^*, y_i^*, F_i^*)\}_{i=1}^n$ and $\mathcal{P} = \{(x_j, y_j, F_j)\}_{j=1}^m$ denote the reference and query peak tables. The assignment cost defined in the main article is augmented to permit unmatched peaks:

$$\widetilde{C} = \begin{bmatrix} C & D_{\text{ref}} \\ D_{\text{qry}} & 0_{m\times n} \end{bmatrix}, \tag{S3}$$

where $D_{\text{ref}} = \text{diag}(U_{\text{ref}})$ and $D_{\text{qry}} = \text{diag}(U_{\text{qry}})$ encode the costs of leaving reference or query peaks unmatched. The bottom-right block allows dummy–dummy assignments

without additional cost. Solving the resulting linear assignment problem with the Hungarian algorithm [5–7] separates real–real matches from missed reference peaks and extra query peaks.

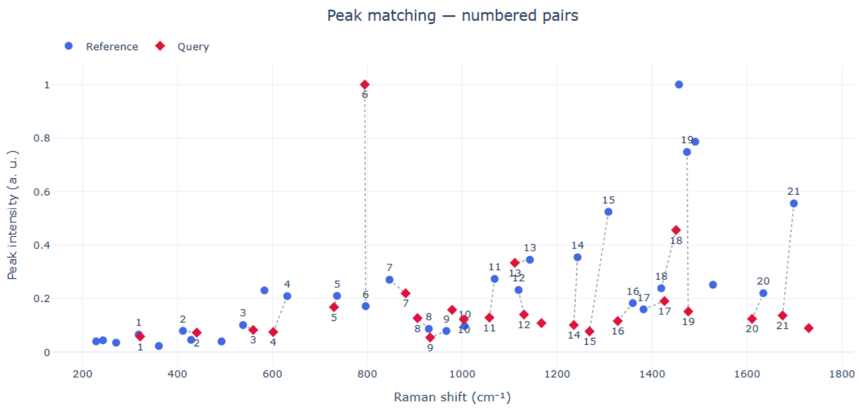

Figure S2. Example Hungarian assignment between reference peaks (blue) and query peaks (red). Connected markers indicate assigned pairs; unconnected markers are treated as orphans.

The assignment and scoring configuration is denoted by

$$\theta_{S_J} = \{\alpha_x, \alpha_y, \tau_{x,\text{match}}, \tau_{y,\text{match}}, \Delta x_{\text{max}}, U_{\text{ref}}, U_{\text{qry}}, \tau_x, \tau_y, \tau_F, \tau_y^{\text{non}}, w\}, \quad \text{(S4)}$$

where w identifies the peak-weighting rule. Table S1 separates parameters used to construct the assignment from those used to score the resulting matches and orphans.

Table S1. Roles of the S_J configuration parameters.

**Parameter**	**Role**
α_x, α_y	Relative contributions of center displacement and normalized intensity difference to the real–real assignment cost.
$\tau_{x,\text{match}}, \tau_{y,\text{match}}$	Scale parameters used in the assignment cost.
Δx_{max}	Physical center gate. Candidate pairs outside this displacement are forbidden.
$U_{\text{ref}}, U_{\text{qry}}$	Costs for leaving a reference or query peak unmatched.
τ_x, τ_y, τ_F	Center, normalized-height, and FWHM tolerances used to score an accepted pair.
τ_y^{non}	Intensity scale used to penalize unmatched peaks. It affects severity and is not a fit-quality threshold.
w	Peak weighting rule. The metric permits user-defined emphasis, and the API exposes uniform and matched-intensity-proportional modes. The benchmark calibration fixes uniform weighting and varies only the tolerances listed in Table S2.

S1.3. S_J parameter sensitivity, calibration, and freezing

The 500 reference identities are divided deterministically within source category into 350 calibration and 150 held-out references, using seed 2027. All 100 perturbations of a reference remain in the same group. This grouping prevents perturbations of one reference from appearing on both sides of parameter selection.

The sensitivity stage operates on the noisy-query peak table associated with every comparison and does not condition on an R^2 threshold. The one-at-a-time grids describe how each scoring tolerance changes the score distribution. A prespecified factorial grid is used for parameter selection. The original configuration is retained as an explicit candidate rather than treated as a privileged result.

Table S2. Prespecified S_J sensitivity and selection grid.

Parameter	Values
One-at-a-time τ_x (cm^{-1})	10, 15, 20, 25, 30, 40, 50
One-at-a-time τ_y	0.10, 0.15, 0.20, 0.25, 0.30, 0.40, 0.50
One-at-a-time τ_F (cm^{-1})	10, 15, 20, 25, 30, 40, 50
One-at-a-time τ_y^{non}	0.050, 0.075, 0.100, 0.125, 0.150, 0.200, 0.250
Factorial τ_x (cm^{-1})	15, 25, 40
Factorial τ_y	0.15, 0.25, 0.40
Factorial τ_F (cm^{-1})	15, 25, 40
Factorial τ_y^{non}	0.075, 0.125, 0.200
Fixed assignment settings during this selection	$\alpha_x = 1$, $\alpha_y = 0.5$, $\tau_{x,\text{match}} = 20$ cm^{-1}, $\tau_{y,\text{match}} = 0.1$, $\Delta x_{\max} = 50$ cm^{-1}, $U_{\text{ref}} = U_{\text{qry}} = 0.5$

For each comparison, the prespecified perturbation burden B is the mean of the percentile ranks of absolute mean shift, absolute mean intensity change, absolute mean FWHM change, numbers of injected, mutated, and dropped peaks, and negative SNR. For a candidate parameter vector θ, the calibration objective is

$$Q(\theta) = -\rho_S\big(S_J(\theta), B\big) + 0.10 \min\left(\frac{\text{IQR}[S_J(\theta)]}{0.20}, 1\right) - 0.25[p_{\leq 0.02}(\theta) + p_{\geq 0.98}(\theta)]. \tag{S5}$$

Thus, selection rewards a monotone decrease with known perturbation burden, retains useful dynamic range, and penalizes saturation near zero or one. It does not maximize the raw S_J magnitude. Ties are resolved deterministically by the stored setting identifier. The four selected tolerances are combined with the fixed assignment settings and uniform peak weighting before any downstream analysis. Perturbation summaries use the complete benchmark with split labels retained, whereas the final retrieval comparison and its bootstrap intervals are reported on held-out reference identities.

The selected scoring tolerances were $\tau_x = 15$ cm^{-1}, $\tau_y = 0.15$, $\tau_F = 15$ cm^{-1}, and $\tau_y^{non} = 0.075$. The fixed assignment settings were $\alpha_x = 1$, $\alpha_y = 0.5$, $\tau_{x,\text{match}} = 20$ cm^{-1}, $\tau_{y,\text{match}} = 0.10$, and $\Delta x_{\max} = 50$ cm^{-1}, with uniform peak weighting. At this setting, the Spearman association between S_J and perturbation burden was -0.522 for the calibration references and -0.489 for the held-out references. Across all comparisons, S_J had a mean of 0.6464 and a median of 0.6577. The perturbation ranges were not restricted to values below the scoring tolerances: the smooth center displacement reached 30 cm^{-1}, twice the selected τ_x. Each tolerance is the half-penalty scale of a smooth sigmoid, so a deviation equal to τ receives a penalty of 0.5 and larger deviations remain admissible while being penalized progressively. The tolerances regulate score sensitivity; they are neither acceptance limits nor favorable perturbation ranges.

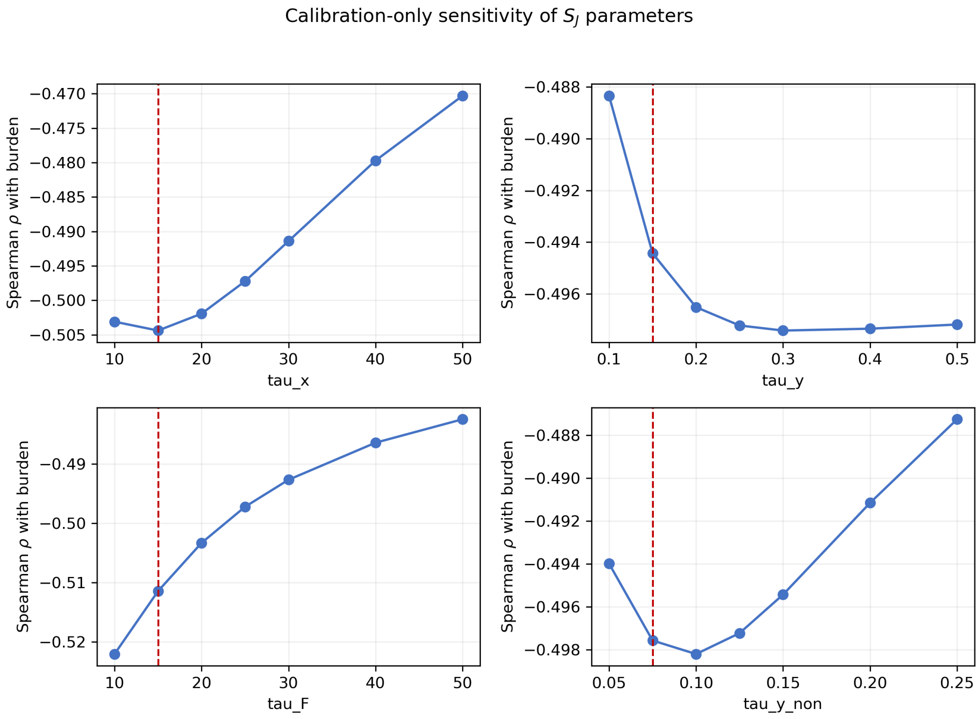

Figure S3. Sensitivity of S_J to the four scoring tolerances in the calibration set. The selected operating point was fixed before held-out evaluation.

S1.4. Lorentzian reconstruction quality

The Lorentzian reconstruction was assessed for every perturbed spectrum by the coefficient of determination between the noisy query and its fitted multi-Lorentzian representation. This assessment describes the preliminary peak table supplied to S_J; it is not an inclusion rule. All 50,000 comparisons were retained for the metric analyses, and no R^2 threshold was applied.

Across the complete benchmark, mean R^2 was 0.9506, the median was 0.9927, and the standard deviation was 0.1129. The 5th and 95th percentiles were 0.6683 and 0.9984, respectively. The lower tail was concentrated at severe noise levels: mean R^2 increased from 0.7209 at SNR 1–3 to 0.9611 at SNR 4–7, 0.9879 at SNR 8–13, 0.9943 at SNR 14–19, and 0.9963 at SNR 20–25.

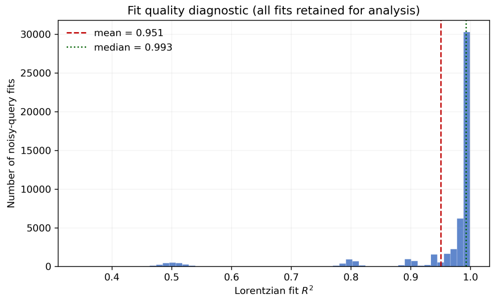

(**a**) Distribution across all comparisons.

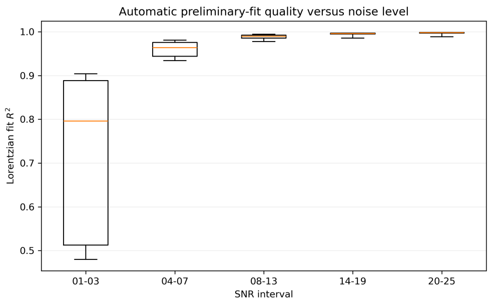

(**b**) Mean R^2 by SNR interval.

Figure S4. Goodness of fit of the automatic multi-Lorentzian representation for the 50,000 perturbed spectra.

S1.5. Perturbation strata and reference-level uncertainty

The frozen metrics are summarized against the continuous perturbation descriptors (mean absolute shift, mean relative intensity change, and mean relative FWHM change), discrete peak events (injection, mutation, and deletion), and SNR. The extended outputs include metric values and losses within prespecified perturbation bins, marginal Spearman associations, adjusted rank associations, SNR-by-split summaries, and the inter-metric Spearman matrix. These analyses compare response patterns across metrics; they do not convert the metrics to a single claim of universal superiority.

All uncertainty analyses use 1,000 deterministic bootstrap replicates. The resampling unit is the reference identity, so the 100 perturbations derived from one reference remain together in every replicate. The pipeline reports bootstrap standard errors and percentile 95% intervals for mean metric values, perturbation-bin losses, and held-out retrieval endpoints. Row-wise resampling is not used because it would treat the 100 dependent perturbations of one parent as independent experimental units.

For S_J, the held-out loss between the lowest- and highest-burden bins was largest for peak-center shift, at 0.2448 (95% CI, 0.2348–0.2541), followed by noise, at 0.1524 (0.1440–0.1616). The corresponding losses were 0.0364 (0.0273–0.0459) for FWHM change and 0.0171 (0.0076–0.0260) for gain. Dropped, mutated, and injected peaks produced losses of 0.0581 (0.0486–0.0687), 0.0292 (0.0208–0.0376), and 0.0286 (0.0235–0.0340), respectively.

Table S3. Held-out S_J loss from the lowest- to highest-burden bin. Intervals are 95% reference-level bootstrap intervals.

Perturbation	Loss	95% CI
Peak-center shift	0.2448	0.2348–0.2541
Inverse SNR	0.1524	0.1440–0.1616
FWHM change	0.0364	0.0273–0.0459
Intensity gain	0.0171	0.0076–0.0260
Dropped peaks	0.0581	0.0486–0.0687
Mutated peaks	0.0292	0.0208–0.0376
Injected peaks	0.0286	0.0235–0.0340

The metrics did not respond identically. Pearson and cosine similarity lost 0.2081 and 0.2171 at the extreme shift bin, whereas cross-correlation and cross-cosine lost only 0.0121 and 0.0099. DTW showed its largest reported loss for noise (0.2123) and no clear extreme-bin shift loss (0.0006, with an interval spanning zero). S_J also remained only moderately correlated with the waveform measures: Spearman correlations ranged from 0.276 to 0.480, compared with 0.877 between cosine and Pearson similarity and 0.908 between cross-correlation and cross-cosine similarity.

S1.6. Full-library retrieval, global complementary weight, and difficult cases

Each perturbed query is searched against all 500 reference identities. The ranking experiment uses cosine similarity and Pearson correlation as fixed-grid measures and cross-cosine similarity and cross-correlation as their rigid-lag counterparts. This paired design isolates the effect of permitting a global shift under angular and correlation-based comparison; DTW and SFEC remain part of the broader perturbation-response analysis. Standalone S_J is also evaluated. Top-k recovery is the proportion of queries for which the known parent appears within the first k ranked references; mean reciprocal rank (MRR) additionally reflects the full position of that parent. Scoring 50,000 queries against all 500

references produces 25,000,000 query–reference comparisons. Individual metric results are reported before any combined result.

Complementarity is evaluated by combining each of these four waveform scores with the frozen S_J value for the same query–reference pair. One global weight is considered at each value from 0 to 1 in increments of 0.05 in $H_w = (1 - w)M_{\text{base}} + wS_J$. The same weight must apply to cosine similarity, Pearson correlation, cross-cosine similarity, and cross-correlation. Selection uses calibration identities only: eligible weights must be non-inferior in calibration MRR for every selector; among them, the rule maximizes the minimum change in MRR across selectors, then the mean change, and finally chooses the smaller weight within an MRR equivalence tolerance of 10^{-4}. Weight zero is retained if no positive weight satisfies the rule. The selected weight is frozen before held-out evaluation.

Difficult cases are classified before interpretation from the full-library correct-parent ranks. The hybrid outcome is recorded as improved, worsened, or unchanged relative to the corresponding standalone waveform selector, with promotion to Top-1 recorded separately. Because every reference receives both components of the hybrid score, there is no candidate-availability category and every query contributes to the summary. Case-level records are generated deterministically from these full-ranking outcomes, and the complete catalog remains in the archive for inspection.

The calibration rule selected $w = 0.50$, and the same value was applied to every selector, candidate, and held-out query. Standalone S_J achieved a held-out Top-1 recovery of 0.9603. The hybrid increased Top-1 recovery from 0.4166 to 0.8828 for cosine similarity, from 0.3960 to 0.9094 for Pearson correlation, from 0.9449 to 0.9930 for cross-correlation, and from 0.9523 to 0.9933 for cross-cosine similarity. The large gains for the fixed-grid measures show that band correspondence recovers information lost under spectral displacement; the smaller but consistent gains for the rigid-lag measures show that S_J also contributes after much of the shift sensitivity has already been removed. Thus, the common weight improves both weaker and already strong rankings without redefining S_J as a replacement for either comparison family. Table S4 reports the complete held-out summary, and the confidence interval for each paired Top-1 change excluded zero.

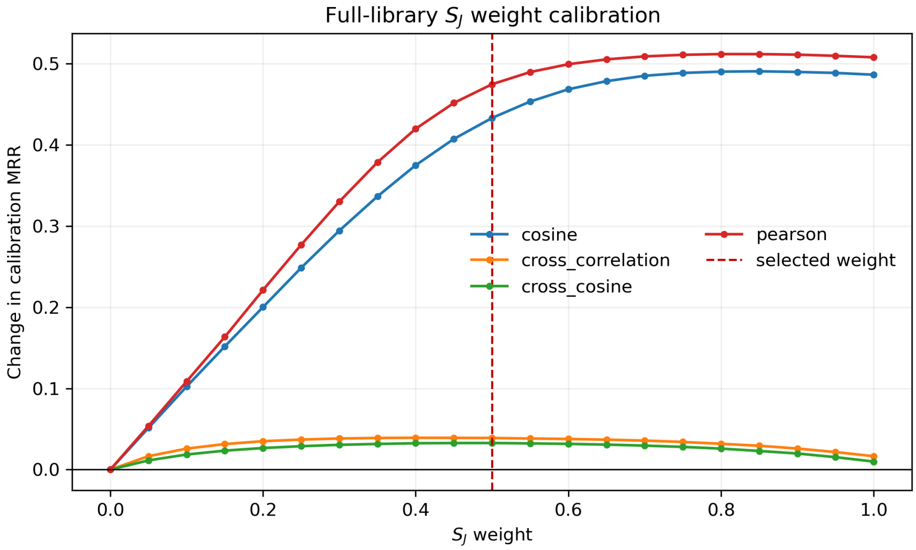

Figure S5. Calibration-set change in mean reciprocal rank across the full 500-reference library as a function of the global S_J weight. The vertical marker identifies the selected value $w = 0.50$.

Relative to the standalone waveform rankings, the hybrid improved the correct-parent rank for 57.8% of cosine queries and 60.0% of Pearson queries, while worsening it for 0.37%

Table S4. Held-out full-library retrieval for the standalone scores and hybrids with $w = 0.50$. Top-k and MRR values are percentages. Confidence intervals refer to the paired Top-1 percentage-point change from the corresponding waveform selector.

Selector	Ranking	Top-1	Top-5	Top-10	Top-20	MRR	Top-1 change (95% CI)
Cosine	Standalone	41.660	57.080	63.960	71.873	49.412	–
	Hybrid	88.280	96.200	98.027	99.160	91.784	46.620 (44.266–48.960)
Pearson	Standalone	39.600	54.887	62.947	73.053	47.499	–
	Hybrid	90.940	97.620	98.740	99.460	93.914	51.340 (48.873–53.607)
Cross-correlation	Standalone	94.487	98.373	99.193	99.580	96.206	–
	Hybrid	99.300	99.893	99.953	99.980	99.568	4.813 (3.820–5.927)
Cross-cosine	Standalone	95.227	98.713	99.333	99.653	96.799	–
	Hybrid	99.327	99.940	99.973	99.980	99.606	4.100 (3.179–5.180)
S_J	Standalone	96.027	98.300	98.780	99.173	97.070	–

and 0.35%, respectively. Cross-correlation and cross-cosine began much closer to the performance ceiling; their ranks improved for 5.39% and 4.64% of queries and worsened for 0.50% and 0.42%. These full-library outcomes quantify both the complementary contribution and the limited tradeoff introduced by the shared hybrid rule.

S1.7. Illustration of shift sensitivity in pointwise metrics

Figure S6 illustrates how modest Raman-shift offsets can reduce Pearson correlation and cosine similarity even when the dominant band pattern remains visually similar. Pointwise metrics compare intensities at identical grid positions. A horizontal displacement near a sharp band center can therefore compare a band maximum in one spectrum with a shoulder in the other, producing an apparently large intensity discrepancy.

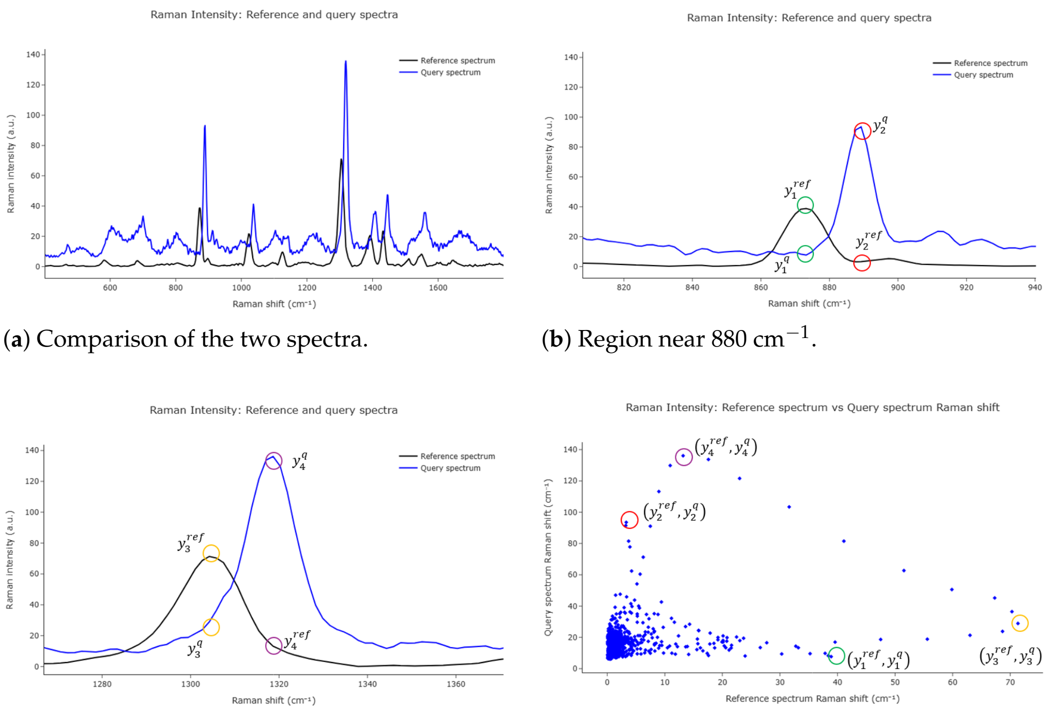

(**a**) Comparison of the two spectra.

(**b**) Region near 880 cm^{-1}.

(**c**) Region near 1310 cm^{-1}.

(**d**) Pointwise intensity scatter.

Figure S6. Illustration of the effect of Raman-band displacement on pointwise similarity metrics.

The enlarged regions show how the displaced samples move away from the $y = x$ line, reducing linear association and dot-product overlap. This figure demonstrates a

mechanism of shift sensitivity; it is not an independent chemical-identification experiment. Alignment-aware and peak-level metrics should therefore be examined alongside pointwise scores when misregistration is plausible [8,9].

S1.8. Hosted API and interactive interface

The Raman similarity service [1] is implemented with FastAPI and provides a documented batch interface and a browser-based interactive workspace. FastAPI publishes the request parameters, accepted file type, response status, and machine-readable OpenAPI schema. Its Swagger interface also permits a request to be executed directly from the browser without writing client code. The public documentation is provided with the service.

An initial request may require a cold-start delay after a period of inactivity. Three principal public POST endpoints are exposed: `/Spectrum_Similarity` for a reference–query batch, `/Lorentz_Fit` for stand-alone peak fitting, and `/Hungarian_Match` for stand-alone peak assignment. The first endpoint can optionally apply baseline removal using the documented IRSQR, ARPLS, or FABC procedure. Baseline removal is disabled by default so that the user makes the preprocessing choice explicitly. The optional `lorentz_tables_file` field of `/Spectrum_Similarity` accepts validated peak tables prepared outside the service; when supplied, those tables replace the automatic fit for the corresponding reference and queries. The GET route `/Paper_Library_Ranking` reconstructs a selected archived noisy query and evaluates it against all 500 reference spectra. Figure S7 shows the public documentation for the deployed API, version 1.2.0.

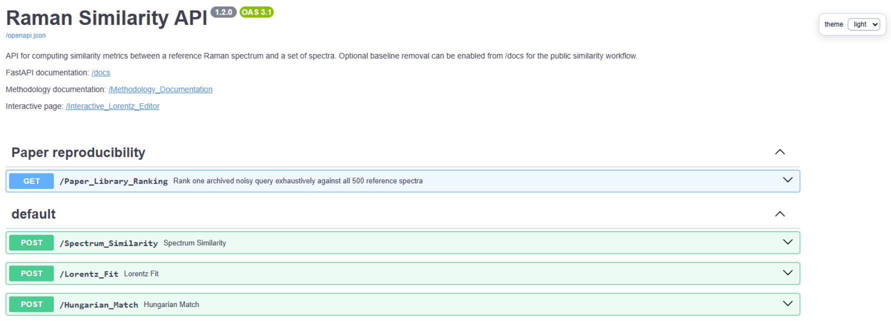

Figure S7. FastAPI/Swagger documentation page for the deployed Raman similarity service. The reproducibility route performs complete 500-reference ranking for an archived query; the three POST routes expose the combined similarity workflow, Lorentzian fitting, and Hungarian peak matching.

The interactive workspace [1] contains two modes. The fitting mode supports inspection and editing of an individual Lorentzian decomposition. The article mode permits inspection of selected reference spectra, perturbed cases, peak tables, provenance, and metric values from the public data package [2]. It first requests a limited catalog and then requests one selected case from the server. The browser therefore does not need to download the complete Zenodo ZIP. A complete library ranking uses the selected query block together with one compact 500-reference library that is cached by the server; all 500 candidates are evaluated by cosine similarity, Pearson correlation, cross-cosine similarity, cross-correlation, and S_J, and the common hybrid weight is applied across the complete candidate set as specified in Section S1.6. The route `/Paper_Interactive_Explorer` redirects to the article mode.

Table S5. Principal user-facing components of the Raman similarity service.

Component	Purpose	Output
API documentation	Batch similarity calculation, standalone Lorentzian fitting, and standalone Hungarian peak matching.	Downloadable workbooks, peak tables, matching diagnostics, and optional figures.
Interactive fitting mode	Inspection and editing of a user-supplied Lorentzian decomposition.	Fitted spectrum, editable peak parameters, candidate models, and exported tables.
Article mode	On-demand inspection of selected objects and cases from the archived benchmark.	Reference and perturbed reconstructions, metric values, peak-table context, and provenance links.
Library-ranking route	On-demand comparison of one archived noisy query with all 500 reference spectra.	Leading candidates for standalone S_J and the four full-library hybrids, including waveform scores and full-library ranks; setting `limit=500` returns the complete order.
Methodology page	Compact reference for equations and implementation parameters.	Metric definitions, tolerance meanings, and parameter defaults.

The batch endpoints process the uploaded spectra on the server and return only the requested workbook, tables, diagnostics, and figures. They do not require the benchmark archive. In article mode, the source spectrum supplies provenance and fitting context, whereas the controlled comparison uses the archived Lorentzian reconstruction and peak tables. The displayed noisy-query fit is the diagnostic representation used by S_J for that archived case. It does not replace the generated perturbed table used to construct the continuous noisy trace evaluated by the waveform metrics. Interactive edits were not used to generate the results reported in the article.

S1.9. Reproducible batch example and Lorentzian review

The `/Spectrum_Similarity` endpoint accepts an `.xlsx` spreadsheet in which the first column contains Raman shift, the second contains the reference intensity, and each remaining column contains a query spectrum on the same grid. Available outputs include Pearson correlation, cosine similarity, SFEC, DTW, cross-correlation, cross-cosine similarity, and S_J. The endpoint performs the requested calculation on demand; a user who wants to compare new spectra does not need the 500-reference archive. An optional second upload can provide externally generated Lorentzian tables as a long-form CSV/XLSX table with a spectrum identifier, or as one XLSX sheet per spectrum. Table identifiers must match the spectral column names. Required quantities are peak center, FWHM or sigma, and height or amplitude; equivalent quantities are derived after validation. A minimal browser-based calculation consists of the following steps:

1. Open the documentation URL, expand `POST /Spectrum_Similarity`, and select `Try it out`.
2. Select the required metrics and optional outputs. If the uploaded spectra have already been baseline corrected, leave `remove_baseline` disabled. Otherwise, enable it and record the selected method. This preprocessing choice must remain consistent across the compared spectra.
3. Use the documented defaults for an exploratory calculation. To reproduce the article configuration, enable `change_default_values` and enter the frozen parameter values stated in Section S1.3. API defaults are not evidence that a parameter value was selected by the article's calibration procedure.
4. Upload a spreadsheet with the column organization illustrated in Figure S8. All spectra must use the Raman-shift grid in the first column.
5. If independently obtained Lorentzian tables are to be used, upload them through `lorentz_tables_file`. Otherwise, leave that field empty and allow the service to produce a preliminary automatic table.
6. Select `Execute`. A completed request reports HTTP status 200 and provides a `Download file` link, as illustrated in Figure S9.
7. Retain the downloaded ZIP together with the input spreadsheet and the selected parameter values. The ZIP contains the requested metric workbook and, when selected, the Lorentzian tables, Hungarian diagnostics, and figures needed to inspect the calculation.

Raman shift (cm-1)	Reference spectrum	Query 1	Query 2	Query 3
200.0	0.002463	0.002578	0.002582	0.001983
206.425703	0.002551	0.002669	0.002671	0.002054
212.851406	0.002643	0.002765	0.002765	0.00213
219.277108	0.002741	0.002866	0.002866	0.00221
225.702811	0.002846	0.002974	0.002972	0.002295
232.128514	0.002956	0.003089	0.003085	0.002385
238.554217	0.003075	0.003211	0.003205	0.002482
244.97992	0.003201	0.003341	0.003334	0.002585
251.405622	0.003335	0.003479	0.003471	0.002695
257.831325	0.00348	0.003628	0.003618	0.002813
264.257028	0.003634	0.003787	0.003775	0.00294
270.682731	0.0038	0.003957	0.003943	0.003077

(**a**) Minimal spreadsheet organization.

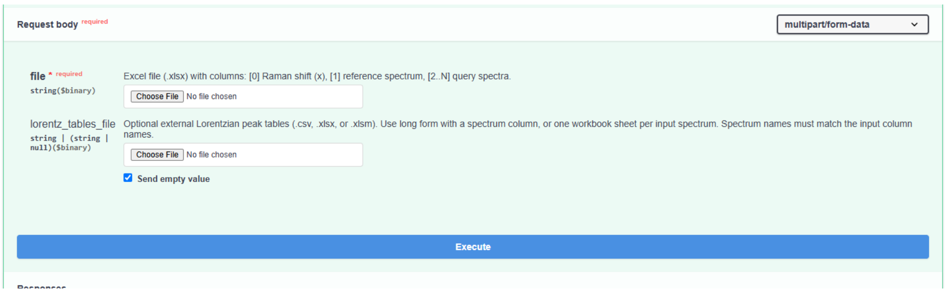

(**b**) Required spectrum file and optional external Lorentzian-table upload fields.

Figure S8. Input preparation and upload for a reproducible batch similarity request. The deployed form accepts the spectrum workbook through `file` and, when independently obtained peak tables are available, accepts them through `lorentz_tables_file`. The file-selector label is supplied by the user's operating system and may therefore appear in the local interface language.

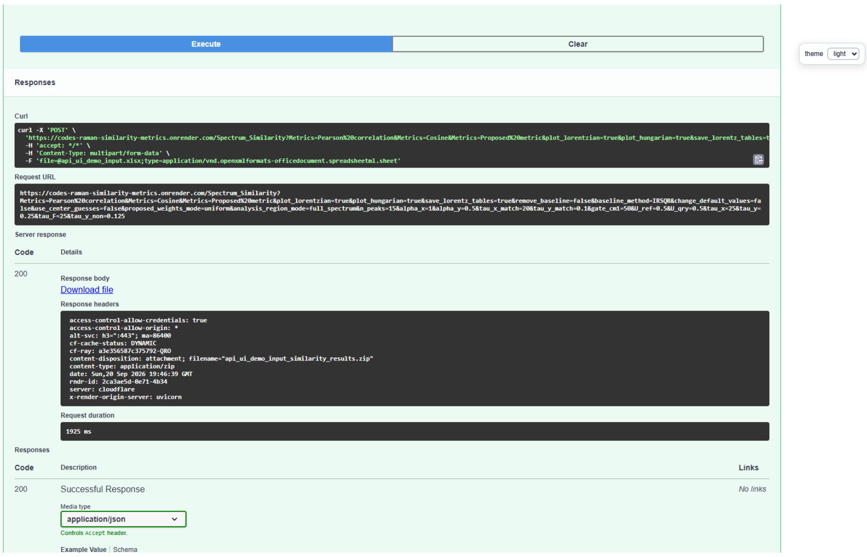

Figure S9. Successful batch request. The server reports status 200 and returns a downloadable ZIP archive; the request URL records the selected metrics and options.

Table S6. Batch input and output structure.

Item	Description
Input spreadsheet	Raman shift in the first column, reference intensity in the second, and one or more query-intensity columns thereafter.
External peak-table file	Optional CSV/XLSX peak tables produced by another validated workflow. Spectrum identifiers must match the input columns; the tables replace automatic fitting for the submitted calculation.
Metric selection	Individual metrics or a combined set may be requested.
Preprocessing choice	Optional baseline removal is available for uploaded data. The chosen method and effective parameters are recorded in the output metadata.
Optional peak outputs	Lorentzian peak tables, reconstructed spectra, Hungarian assignments, orphan counts, and per-pair match-quality diagnostics.
Download package	A compressed archive containing the requested numerical results and any selected diagnostic figures or peak tables.

Manual review of the Lorentzian proposal

The interactive fitting mode accepts `.xlsx`, `.csv`, `.txt`, and `.dat` spectra and first computes an automatic Lorentzian proposal. That proposal is intentionally editable. A user can drag the yellow apex handles to revise peak center or height, drag the cyan handles to revise FWHM, or enter center, height, and FWHM values directly in the editable rows. Individual peaks can be enabled, disabled, deleted, or added; candidate models selected by BIC or AIC remain visible for comparison. `Undo` and `Redo` preserve reversible inspection.

After a manual change, the interface labels the displayed state `manual_preview`. Selecting `Refit edited rows` optimizes from the edited values and produces an optimized_refit; selecting `Compute automatic fit` again replaces the edits with a new `automatic_fit`. The

displayed table can be exported as CSV or JSON. Figure S10 shows a manual preview after increasing the first peak center by 2 cm^{-1} relative to the automatic proposal.

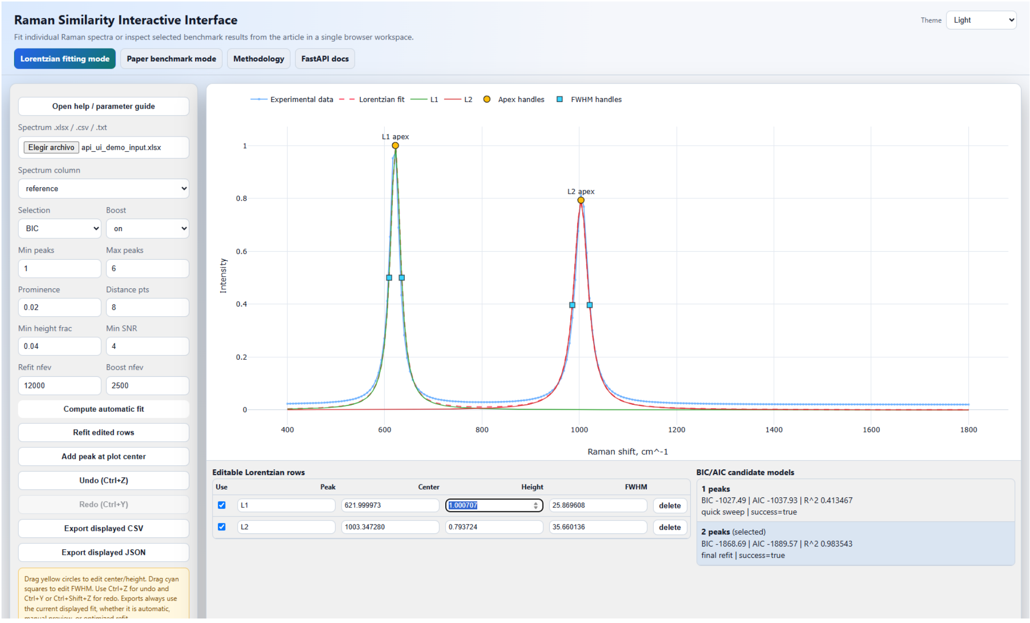

Figure S10. Interactive Lorentzian fitting mode after a manual center edit. Yellow circles control center and height, cyan squares control FWHM, and the table permits direct numerical editing. Candidate models and export controls remain available during review.

Manual editing is provided for transparent expert inspection and user-supplied analyses. Exported CSV or JSON records preserve whether the displayed state is an automatic fit, an unoptimized manual preview, or an optimized refit. This distinction prevents a manually moved curve from being mistaken for an automatic result. Manual editing was not used to generate, tune, or replace any Lorentzian table in the reported benchmark; all article results were produced by the frozen automated workflow.

S1.10. Benchmark composition and archived evidence

The final benchmark contains 500 unique reference spectra and 100 perturbed realizations per reference, yielding 50,000 reference–query comparisons. The source composition is shown in Table S7. RamanSPy entries are synthetic; the remaining 249 records are public experimental or reference spectra. The public/reference records originate from RRUFF [10], NASA RAMdb [11], the OpenSpecy library data [12], and the Raman Open Database [13]; the synthetic records were generated with RamanSPy [14]. Fourteen duplicated OSU/OpenSpecy sample keys were replaced by unique OSU records while preserving the final source counts. Available acquisition and sample metadata were retained as reported by the source repositories. Physical state, sample form, and grain size were not reported consistently; these attributes were therefore neither inferred nor used as statistical covariates.

Table S8. Principal evidence classes in the benchmark archive.

Archive component	Scientific role
Traceability and source-citation records	Identify the 500 spectra, source categories, available links, spectral ranges, file-integrity identifiers, and source-to-benchmark mappings.

Archive component	Scientific role
Experimental-condition metadata	Preserve reported acquisition fields and mark unavailable information as missing rather than inferred.
Reference spectra and Lorentzian tables	Provide the source traces and fitted reference objects used in the controlled benchmark.
Generated perturbed peak tables	Record the peak-level realizations used to reconstruct the 50,000 perturbed spectra.
Noisy-query fit tables	Record the fitted table used by S_J for every noisy perturbed spectrum and support the reported R^2 summaries.
Metric and perturbation tables	Provide one row per comparison together with the corresponding perturbation descriptors and reconstruction identifiers.
Calibration and held-out records	Preserve the grouped split, complete parameter grids, selected θ_{S_J}, and global library-weight selection.
Uncertainty, sensitivity, fit-quality, and retrieval outputs	Support the reported confidence intervals, parameter sweeps, R^2 summaries, ranking analyses, and difficult-case classifications.
Figures and provenance records	Provide plotted assets, data dictionaries, source records, manifests, and checksums.

The benchmark uses global seed 2027 and an SNR range of 1 to 25. The complete archive, `raman_similarity_metrics_benchmark.zip`, stores the 500 references, reference and generated perturbed peak tables, every noisy-query fit table, the complete 50,000-row metric table, the grouped calibration and held-out assignment, sensitivity and retrieval outputs, difficult-case records, bootstrap summaries, the frozen configuration, and file-level SHA-256 checksums. The public metric table reports R^2 without an acceptance category or exclusion flag. The dataset archive contains scientific data products and documentation; the implementation used by the online service is maintained separately and is not duplicated as a collection of historical fitting programs.

The 50,000 continuous noisy-query arrays are not stored as separate files. They can be reconstructed numerically from the spectral grid, the perturbed Lorentzian peak table, SNR, and the archived PCG64 state immediately preceding the Gaussian draw. The local seed alone is insufficient because random numbers are consumed during earlier perturbation steps. For this reason, the archive includes the per-case pre-noise generator state, the reconstruction specification, runtime-version metadata, and validation records covering all 50,000 cases. This representation reduces transfer size without removing the information needed to regenerate the noisy trace used by the waveform metrics.

For efficient online use, the Zenodo record [2] provides the complete archive, a checksum-protected online_manifest.json, a compact shared file named raman_similarity_metrics_library.zip, and 20 independently downloadable data blocks. The shared file contains all 500 reference spectra and their benchmark peak representations and is cached once for complete library ranking. Each block contains 25 references and their 2500 perturbed comparisons, including reference and query peak tables, perturbation descriptors, final metric rows, retrieval rows, R^2 values, and noise-reconstruction states. A selected-case request downloads only its block; a full ranking uses that block plus the compact shared

Table S7. Source composition of the final 500-spectrum benchmark.

Source category	Spectra
RamanSPy-generated synthetic spectra	251
RRUFF	145
Raman Open Database	55
NASA RAMdb	17
OpenSpecy	17
OSU	15
Total	500

library. The complete archive is reserved for bulk download. The selective files reproduce the same scientific records as the complete archive.

The dataset version supporting this article is published on Zenodo [2]. The cited DOI resolves directly to the 23-file archive used by the hosted API and described above.

S1.11. Interpretation and transfer limits

Source spectra preserve provenance and empirical morphology, whereas the controlled calculations use fitted peak tables and their reconstructions. The shared Lorentzian representation used to generate perturbations and evaluate S_J should therefore remain explicit when the structural score is interpreted.

The noisy-query fit estimates the peak table visible in a noisy realization, whereas the waveform metrics operate on continuous traces sampled on a common grid. The two representations therefore characterize different evidence from the same query.

The retrieval experiment is a controlled parent-spectrum recovery test. It evaluates recovery of the known reference from the 500-spectrum library and the contribution of S_J in full-library hybrid rankings; it is not a blind chemical-identification benchmark with curated near-neighbor distractors.

Validation of the workflow for another Raman modality or instrument remains future work and will require application-specific preprocessing, peak extraction, tolerance calibration, and representative data. This requirement is particularly important for broad or congested biological spectra, SERS, hyper-Raman measurements, strong fluorescence or nonlinear backgrounds, and acquisition conditions not represented here. For broad, overlapping, or otherwise difficult band structures, the peak table may be established by expert curation, specialized instrument software, or another validated decomposition instead of the automatic proposal used in this benchmark. Tolerances, peak weights, lag windows, excluded spectral regions, and peak-table provenance should be reported as part of the comparison protocol. Although S_J already accepts alternative peak weights, the effects of diagnostic-band or region-specific weighting were not evaluated in this benchmark and remain a subject for future application-specific studies. The benchmark does not model laser-heating dynamics, non-stationary scattering, Doppler shifts, grain-size effects, or missing low-shift measurements as separate physical mechanisms. Its controlled Raman-shift displacement is phenomenological, and interpretation below 200 cm^{-1} remains conditional on the source spectrum's measured range and instrument filters.

We did not reimplement external peak-aware methods as surrogate baselines because their preprocessing, peak-selection, region-definition, and calibration procedures have not been validated for this heterogeneous benchmark. A direct cross-method evaluation will require validated implementations, common evaluation data, and application-appropriate calibration rules.

1. Raman Similarity Metrics API, version 1.2.0. Online service.
2. A. Reyes-Reyes, *Raman Similarity Metrics Benchmark: Spectra, Perturbations, and Evaluation Results*, Zenodo. doi:10.5281/zenodo.22958814.
3. A. Savitzky and M. J. E. Golay, *Smoothing and differentiation of data by simplified least squares procedures*, Analytical Chemistry, 36 (1964) 1627–1639. doi:10.1021/ac60214a047.
4. M. Wojdyr, *Fityk: a general-purpose peak fitting program*, Journal of Applied Crystallography, 43 (2010) 1126–1128. doi:10.1107/S0021889810030499.
5. H. W. Kuhn, *The Hungarian method for the assignment problem*, Naval Research Logistics Quarterly, 2 (1955) 83–97. doi:10.1002/nav.3800020109.
6. H. W. Kuhn, *Variants of the Hungarian method for assignment problems*, Naval Research Logistics Quarterly, 3 (1956) 253–258. doi:10.1002/nav.3800030404.
7. R. E. Burkard and E. Cela, *Linear assignment problems and extensions*, in: Handbook of Combinatorial Optimization, Springer, Boston, MA, 1999, pp. 75–149. doi:10.1007/978-1-4757-3023-4_2.
8. S. Mostafapour, T. Dörfer, R. Heinke, P. Rösch, J. Popp, and T. Bocklitz, *Investigating the effect of different pre-treatment methods on Raman spectra recorded with different excitation wavelengths*, Spectrochimica Acta Part A, 302 (2023) 123100. doi:10.1016/j.saa.2023.123100.
9. S. Mostafapour, A. Mokari, S. Guo, J. Popp, and T. Bocklitz, *Raman spectra comparison: cautions and pitfalls of similarity metrics*, Spectrochimica Acta Part A, 344 (2025) 126702. doi:10.1016/j.saa.2025.126702.
10. B. Lafuente, R. T. Downs, H. Yang, and N. Stone, *The power of databases: the RRUFF project*, in: T. Armbruster and R. M. Danisi (Eds.), Highlights in Mineralogical Crystallography, De Gruyter, Berlin, Germany, 2015, pp. 1–30. doi:10.1515/9783110417104-003.
11. A. L. Mattioda, L. Gavilan, C. L. Ricketts, P. K. Najeeb, A. Ricca, and C. Boersma, *The NASA Raman spectroscopic database: RAMdb version 1.00*, Icarus, 408 (2024) 115769. doi:10.1016/j.icarus.2023.115769.
12. OpenSpecy, *OpenSpecy reference library data*, Open Science Framework. Data repository.
13. Raman Open Database, *Open-access Raman spectral database developed for the SOLSA project*. Data repository.
14. D. Georgiev, S. V. Pedersen, R. Xie, C. Fernández-Galiana, M. M. Stevens, and M. Barahona, *RamanSPy: An open-source Python package for integrative Raman spectroscopy data analysis*, Analytical Chemistry, 96 (2024) 8492–8500. doi:10.1021/acs.analchem.4c00383.
